# Comparison of Bayesian Models to Estimate Survival From Dead‐Recovery Alone and Together With Live‐Encounter Data: Challenges and Opportunities

**DOI:** 10.1002/ece3.71517

**Published:** 2025-06-01

**Authors:** Michael Schaub, Jaume A. Badia‐Boher

**Affiliations:** ^1^ Schweizerische Vogelwarte Sempach Switzerland

**Keywords:** band‐recovery, Bayesian, capture–recapture, marginalized, multinomial model, multistate state‐space model, survival

## Abstract

The recovery of dead marked individuals, either alone or in combination with encounters of these individuals while alive, is an important source of data for estimating survival in birds, mammals, and fish. Various models have been developed to analyze such data in a Bayesian framework, including single‐state and multistate state‐space models, marginalized state‐space models, and multinomial models. An overview of the different formulations, together with an assessment of their parameter accuracy, computational efficiency, and flexibility in covariate modeling, is lacking so far. We assessed 13 models based on data simulation and analysis with the widely used R‐based software NIMBLE and JAGS. We found that all the models evaluated produced accurate parameter estimates, with the exception of the multistate state‐space models, which produced biased parameter estimates. This is because the standard MCMC samplers required for Bayesian inference do not work properly for this model. Although such multistate models work correctly in the frequentist framework, they should not be used in the Bayesian framework unless specially developed samplers are used. Instead, single‐state state‐space models, marginalized multistate state‐space models, multinomial multistate models, or reparameterized multistate models should be used. The marginalized state‐space and multinomial models were the most computationally efficient. The models evaluated do not differ in their ability to model temporal covariates but do differ for individual continuous covariates. The latter can be modeled in state‐space models but not in multinomial models. We also show that single‐state models can be formulated for the joint analysis of dead‐recovery and live encounter data, which are usually modeled with multistate models. This facilitates the inclusion of further auxiliary data and results in a computationally efficient model. We expect our overview to help ecologists decide which model to use when estimating survival from dead‐recovery data in the Bayesian framework.

## Graphical abstract

Different models in the Bayesian framework for analyzing dead‐recovery data, either alone or together with live‐encounter data to estimate survival, are presented and compared. Although all but one of the models provide unbiased results, they differ in their computational efficiency and the way in which covariates can be included.

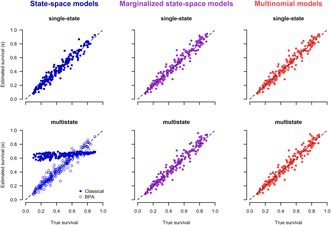

## 1 Introduction

Survival is a key demographic parameter that plays a crucial role in population dynamics (Sæther and Bakke 2000). Estimating survival probabilities essentially requires tracking individuals over time, and the proportion of individuals that have survived over a given time interval is an estimate of survival at that time resolution. Although there are models that allow survival to be estimated from count data without knowledge of individual identities (Dail and Madsen 2011; Bellier et al. 2016), the most commonly used approaches rely on individually identifiable animals (through artificial markings or using natural characteristics of the individuals) that are released and may be encountered alive or found dead later (Brownie et al. 1985; Lebreton et al. 1992). Because detection of living and dead individuals is usually imperfect, several statistical models have been developed that explicitly estimate detection along with survival probabilities (Williams et al. 2002).

One important source of information about survival is the recovery of dead individuals that have previously been marked. These data are known as dead‐recovery, ring‐recovery, band‐recovery, or mark‐recovery data and are often available for birds (Du Feu et al. 2016), but increasingly for mammals (Hall et al. 2001; Servanty et al. 2010) and fish (Hostetter et al. 2018; Nater et al. 2020) as well. Statistical models for analyzing dead‐recovery data were developed in the 1950's (Darroch 1958; Seber 1970; Brownie et al. 1985) and aim to simultaneously estimate the probability of survival and the probability of recovering a dead, marked individual. Traditional models for parameter estimation used the multinomial likelihood (Brownie et al. 1985). Later, it was recognized that estimation is also possible using multistate capture–recapture models with the states alive and dead (Lebreton et al. 1999).

The second main source of information for estimating survival is repeated encounters of marked individuals that are still alive, known as capture–recapture or live‐encounter data. The first statistical methods for analyzing such data were developed in the 1960's (Cormack 1964; Jolly 1965; Seber 1965), and are known as Cormack‐Jolly‐Seber (CJS) models (Lebreton et al. 1992). Since both dead‐recovery data and live‐encounter data contain information about survival, a logical next step was to develop models that jointly analyze these data (Szymczak and Rexstad 1991; Burnham 1993). The resulting model requires two re‐encounter parameters, one for individuals that are recovered dead and one for individuals that are encountered alive. The joint analysis can again be formulated using a multinomial likelihood (Szymczak and Rexstad 1991, Burnham 1993), or using the likelihood of a multistate capture–recapture model (Lebreton et al. 1999).

The Bayesian mode of analysis for estimating survival from dead recovery and/or live‐encounter data (Brooks, Catchpole, and Morgan 2000; Brooks, Catchpole, Morgan, and Barry 2000; Brooks et al. 2002; King et al. 2010; Kéry and Schaub 2012; King 2012) is used increasingly often (Brooks 2003). The Bayesian mode offers some advantages over the frequentist mode of analysis; in particular, it facilitates the inclusion of random effects and prior knowledge about the estimates (Barry et al. 2003; Lemoine 2019). It also allows the straightforward formulation of state‐space models to analyze the data, which is appealing because it reflects the emergence of the data as a hierarchical process with an underlying latent survival process and a conditional and partially imperfect observation process. However, Kéry and Schaub (2012) realized that the application of the multistate model suggested by Lebreton et al. (1999) to estimate survival jointly from dead‐recovery and live‐encounter data leads to biased estimates. They did not provide an explanation as to why estimates were biased when the frequentist analysis of the same model worked correctly, but proposed a re‐parameterization of the model as a solution (see Methods).

Several different models, such as single‐ and multistate state‐space models, marginalized versions of them, and multinomial models, can be used to analyze dead recovery data, either alone or together with live‐encounter data, using Bayesian methods, but an overview of these and an assessment of the benefits and challenges of each is lacking so far. The aim of this paper is to provide such an overview. We present different models, assess the performance of their estimators and their computational efficiency, and discuss their flexibility in covariate modeling or extensions. This should help users to choose which approach is likely to be the best for their data and research questions.

## 2 Material and Methods

We use simulations to evaluate the performance of the models studied. We begin by describing the different models, firstly for analyzing dead recovery data alone, and secondly for analyzing dead recovery data jointly with live‐encounter data. We then describe how we simulated the data and how we assessed the performance of the models.

### 2.1 Description of Models for Dead‐Recovery Data Alone

We assume that each year several individuals are marked and released into the wild, and that some of these individuals are found dead. The data collected are summarized in individual detection histories, which is a matrix containing 0's and 1's, and denoted as ***y***. The number of rows corresponds to the number of individuals marked and the number of columns to the number of encounter occasions (often years). The occasion of marking of each individual *i* (*f*_*i*_) is indicated by a 1 in the detection history (*y*_*i,f*(*i*)_ = 1). If the individual *i* died and is recovered between occasion *t* − 1 and *t*, there is an additional 1 at occasion *t* (*y*_*i,t*_ = 1). All other elements of the detection history matrix ***y*** are 0's. An important assumption is that individuals that are found dead between occasion *t* − 1 and *t* have died during that time interval. Model extensions that allow the inclusion of recoveries of individuals that died before the time interval in which they were recovered are possible (Catchpole et al. 2001; King 2012), but are not considered here. The basic parameters to be estimated are *s*_*t*_, the probability to survive from occasion *t* to occasion *t* + 1 (if the interval is 1 year, then we estimate annual survival), and *r*_*t*_, the probability that an individual that has died between occasions *t* and *t* + 1, is recovered. This corresponds to the so‐called Seber parameterization of the dead‐recovery model (Seber 1970). Other parameterizations that decompose the recovery probability or use the direct recoveries are also possible (Brownie et al. 1985; Williams et al. 2002), but are not considered here. Furthermore, for simplicity, we here only consider models with time‐constant parameters (i.e., *s* = *s*_t_, *r* = *r*_t_), but the models can also be easily formulated with parameters that vary over time or among individuals.

We present several different models for estimating survival and recovery parameters from the data. The models can be developed for single or multiple states, and each can be analyzed by explicitly estimating the latent states, i.e., using a state‐space approach, or without estimating the latent state, using a multinomial model or a marginalized state‐space approach.

#### 2.1.1 Single‐State State‐Space Model

We introduce the latent state matrix ***z***, which denotes the true state of all individuals at all occasions: it is *z*_*i,t*_ = 1 if individual *i* is alive at occasion *t* and *z*_*i,t*_ = 0 if it is dead. We also define the vector *f*_*i*_ containing the occasion on which individual *i* is marked. When an individual is marked, it is alive and its state is known. On subsequent occasions, the latent state is estimated from the Bernoulli distribution with probability *s*. If the individual dies between *t* and *t* + 1, the latent state changes from *z*_*i,t*_ = 1 to *z*_*i,t*+1_ = 0, and the individual can be recovered with probability *r*, which is modeled using the observed data ***y*** with another Bernoulli trial. Mathematically, the model is written as:

(1) $$\begin{array}{l} z_{i,f_{i}}=1\\ z_{i,t+1}∼\text{Bernoulli}\left(z_{i,t}s\right)\\ y_{i,t}∼\text{Bernoulli}\left(\left(z_{i,t-1}-z_{i,t}\right)r\right) \end{array}$$

#### 2.1.2 Multistate State‐Space Model

To estimate survival using a multistate model, we define a latent state matrix ***z*** with three different levels depending on whether the individual is ‘alive’ (*z*_*i,t*_ = 1), ‘recently dead’ (*z*_*i,t*_ = 2) or ‘long dead’ (*z*_*i,t*_ = 3) (Lebreton et al. 1999). An individual that dies between *t* − 1 and *t* first transitions from state ‘alive’ to the state ‘recently dead’ at *t*, and second to the state ‘long dead’ at *t* + 1 where it remains until the end of the study. The state ‘long dead’ is unobservable and absorbing. It seems odd to have two states for a dead individual, but this is necessary to ensure that (1) an individual can only be recovered in the interval in which it died, and (2) a dead individual can only be recovered once.

The original capture history matrix ***y*** needs to be modified in order to be analyzed with the multistate model. The detection history now needs to distinguish between the observed states ‘marked’ (1), ‘recovered dead’ (2) and ‘not encountered’ (3). Therefore, all 0's in the original ***y*** are replaced by 3's and the dead recoveries indicated by 1's in ***y*** must become 2's in the multistate capture history, denoted ***v***.

Key elements of the multistate model are the state‐transition matrix (Ω) which describes the development of the latent states over time, and the observation matrix (Θ) which links the latent states to the observations. The state‐transition matrix (Ω) contains the probabilities that a latent state at occasion *t* (in the rows) transitions to a latent state at occasion *t* + 1 (in the columns):

(2) $$\Omega =\left[\begin{array}{ccc} s & 1-s & 0\\ 0 & 0 & 1\\ 0 & 0 & 1 \end{array}\right]$$

The observation matrix (Θ) contains the probabilities that the latent states (in the rows) at *t* result in an observed state (in the columns) at *t*:

(3) $$\Theta =\left[\begin{array}{ccc} 0 & 0 & 1\\ 0 & r & 1-r\\ 0 & 0 & 1 \end{array}\right]$$

From the observation matrix it is clear that individuals in latent states 1 (alive) and 3 (long dead) are ‘not encountered’ with probability 1. Therefore, only individuals in latent state 2 (recently dead) can be encountered with probability *r*.

The implementation of this model as a state‐space model requires the use of categorical distributions whose parameters are elements of either the transition or observation matrix. The state at marking is known, but state transitions and the link between the true and the observed states are modeled using the following relationships:

(4) $$\begin{array}{l} z_{i,f_{i}}=1\\ z_{i,t+1}∼\mathrm{categorical}\left(\Omega_{z_{i,t},1...3}\right)\\ v_{i,t}∼\mathrm{categorical}\left(\Theta_{z_{i,t},1...3}\right) \end{array}$$

To clearly distinguish this model from the following one, we name it ‘classical’ multistate state‐space model.

#### 2.1.3 Multistate State‐Space Model, BPA Variant

Kéry and Schaub (2012) found that the multistate state‐space model (section 2.1.2.) produces biased parameter estimates when fitted in the Bayesian framework. They proposed the use of a modified multistate state‐space model, in which the recovery process is included in the state process, and which uses a different definition of the latent states. The three latent states are now ‘alive’ (1), ‘recently dead and recovered’ (2), and ‘recently dead and not recovered, or long dead’ (3). The state transition matrix (Ω) becomes then

(5) $$\Omega =\left[\begin{array}{ccc} s & \left(1-s\right)r & \left(1-s\right)\left(1-r\right)\\ 0 & 0 & 1\\ 0 & 0 & 1 \end{array}\right]$$

The observed states (1: ‘marking’, 2: ‘recovered dead’, 3: ‘not encountered’) are the same as in the original multistate model, but the observation matrix (Θ) becomes completely deterministic:

(6) $$\Theta =\left[\begin{array}{ccc} 0 & 0 & 1\\ 0 & 1 & 0\\ 0 & 0 & 1 \end{array}\right]$$

The implementation as a state‐space model is done in the same way as for the previous model using Equation (4), and the data are also the same (***v***).

#### 2.1.4 Marginalized Single‐State State‐Space Model

The single‐state state‐space model of Section 2.1.1 can be marginalized (Yackulic et al. 2020). Marginalized approaches do not estimate the true latent state, but rather calculate the probability of an individual capture history based on the underlying parameters, here survival and recovery. For instance, if an individual capture history is “10” (marked at occasion 1, then missed at occasion 2), the individual could either have died without being recovered at occasion 2 with probability (1 − *s*)*(1 − *r*), or have survived with probability *s*. The probability of observing this capture history is then (1 − *s*)*(1 − *r*) + *s*. Probability expressions will be considerably longer as capture histories include more occasions. The sum of the capture history probabilities across all individuals gives the probability of the data. This approach, including an algorithm to calculate the probabilities of capture histories, is described in further detail in Yackulic et al. (2020).

Usually, many individuals have identical capture histories—for example, many individuals may be marked at the same occasion but never recovered. The size of the dataset can be reduced by using only unique capture histories (Yackulic et al. 2020). The likelihood of capture histories that originally occurred multiple times in the data then only needs to be calculated once. Their contribution to the data likelihood must be weighted by the frequency of the corresponding capture history. Using this approach, which we call the marginalized single‐state state‐space model for pooled data, we do not expect to find different parameter estimates from unpooled data, but we have included it here to assess the gain in computational efficiency.

#### 2.1.5 Marginalized Multistate State‐Space Model

The multistate state‐space model of Section 2.1.2 can be marginalized in an analogous way as the single‐state state‐space model. Here the state‐transition matrix (Equation (2)) and the observation matrix (Equation (3)) are used to calculate the probability of each capture history in data ***v***. We also fitted this model to the pooled data to gauge the gain in computation efficiency.

#### 2.1.6 Single‐State Multinomial Model

The multinomial likelihood is the classical way of analyzing dead‐recovery data (Brownie et al. 1985; Williams et al. 2002). Individual encounter histories (***y***) are summarized in so‐called m‐arrays (***m***), which count how many individuals marked on a given occasion *t* were recovered on each of the subsequent occasions {*t* + 1, *t* + 2, …,*T*}, and how many were not recovered until the final occasion *T*. The total number of individuals marked at *t* is *R*_t_, and the probabilities of recovering individuals on each of the subsequent occasions, or of not recovering any ($\pi_{t}$) can be expressed as a function of the survival and recovery probabilities (Table 1). This allows the application of the multinomial distribution, such that:

(7) $$m_{t,1...T+1}∼\text{Multinomial}\left(\pi_{t,1...T+1},R_{t}\right)$$

**TABLE 1:** Schematic for the probabilities in the m‐array for the dead‐recovery model with 4 occasions. The parameters to express the cell probabilities are survival (*s*) and recovery (*r*) probabilities. The probabilities of the m‐array are stored in the 3 × 4 matrix π.

Rel. occ	Recovered on occasion			Never recovered
	2	3	4	
1	(1 − *s*)*r*	*s*(1 − *s*)*r*	*ss*(1 − *s*)*r*	(1 − *s*)(1 − *r*) + *s*(1 − *s*)(1 − *r*) + *ss*(1 − *s*)(1 − *r*) + *sss* = 1 − Σ(rel. occ 1)
2	0	(1 − *s*)*r*	*s*(1 − *s*)*r*	(1 − *s*)(1 − *r*) + *s*(1 − *s*)(1 − *r*) + *ss* = 1 − Σ(rel. occ 2)
3	0	0	(1 − *s*)*r*	(1 − *s*)(1 − *r*) + *s* = 1 − Σ(rel. occ 3)

#### 2.1.7 Multistate Multinomial Model

The multinomial likelihood also exists for multistate models (Williams et al. 2002). The individual detection histories (***w***, is the same as ***v***, but all ‘3’ are replaced by ‘0’) are summarized in a multistate m‐array (***n***). It summarizes the number of individuals released in state *j* on occasion *t* that are next encountered in state {*j*, …, *J*; where *J* is the number of states} on occasions {*t* + 1, *t* + 2, …, *T*}; $n_{t,k}^{j,i}$ is the number of individuals released at *t* in state *j* that are first encountered at *k* in state *i*. The probabilities to reencounter individuals at particular occasions and states is again expressed as a function of survival and recovery probabilities.

The m‐array cell probabilities ($\tau_{t}$) are calculated by matrix multiplication of a state transition matrix and an observation matrix (Williams et al. 2002; Schaub and Kéry 2022). The state transition matrix (Ω) is the same as shown in Equation (2), but the observation matrix has the state‐specific probability of encountering an individual as the diagonal and is zero otherwise:

(8) $$\Gamma =\left[\begin{array}{ccc} 0 & 0 & 0\\ 0 & r & 0\\ 0 & 0 & 0 \end{array}\right]$$

From the multistate m‐array the number of released individuals for each occasion *t* and state *j* is calculated ($R_{t}^{j}$). The data are then modeled using the multinomial likelihood as:

(9) $$n_{j,1...J}^{t,1...T+1}∼\text{Multinomial}\left(\tau_{j,1...J}^{t,1...T+1},R_{j}^{t}\right)$$

### 2.2 Description of Models for Joint Analysis of Dead‐Recovery and Live‐Encounter Data

Individuals are marked and may be encountered alive and/or found dead on subsequent occasions. Live encounters usually occur within a defined study area during specific and typically short periods of time. Encounters are broadly defined and may involve physical capture of the individual, or identification from a distance (resighting). The origin of the dead recoveries is not restricted to a specific area. Under certain assumptions, the spatial mismatch between recoveries and live encounters can be used to estimate fidelity to the study area in addition to survival (Burnham 1993). Here we do not consider this, but assume that both live encounters and dead recoveries are from the same area. The data collected are again stored in individual capture histories and all of them comprise the data matrix ***y’***, but now we need different labels to distinguish a live encounter from a dead recovery. We use a 1 each time an individual is encountered alive, a 2 when the individual is found dead, and a 0 otherwise. The basic parameters to be estimated are survival (*s*) and recovery (*r*) as before, but in addition we need the probability of encountering a live marked individual (*p*).

As before, we present several different models for estimating survival, live encounter and recovery parameters from the data. The models can be developed for single or multiple states, and each can be analyzed by explicitly estimating the latent states, i.e., using a state‐space approach, or without estimating the latent state, using a multinomial model or marginalized approaches.

#### 2.2.1 Single‐State State‐Space Model

For each individual *i* and occasion *t* we define the latent state ***z***, denoting whether *i* is alive at *t* (*z*_*i,t*_ = 1) or dead (*z*_*i,t*_ = 0). Individual *i* is alive at the marking occasion *f*_*i*_, and hence

$$z_{i,f_{i}}=1$$

At subsequent occasions, the latent state is modeled as a Bernoulli trial governed by the survival probability *s*:

$$z_{i,t+1}∼\text{Bernoulli}\left(z_{i,t}s\right).$$

**FIGURE 1:**
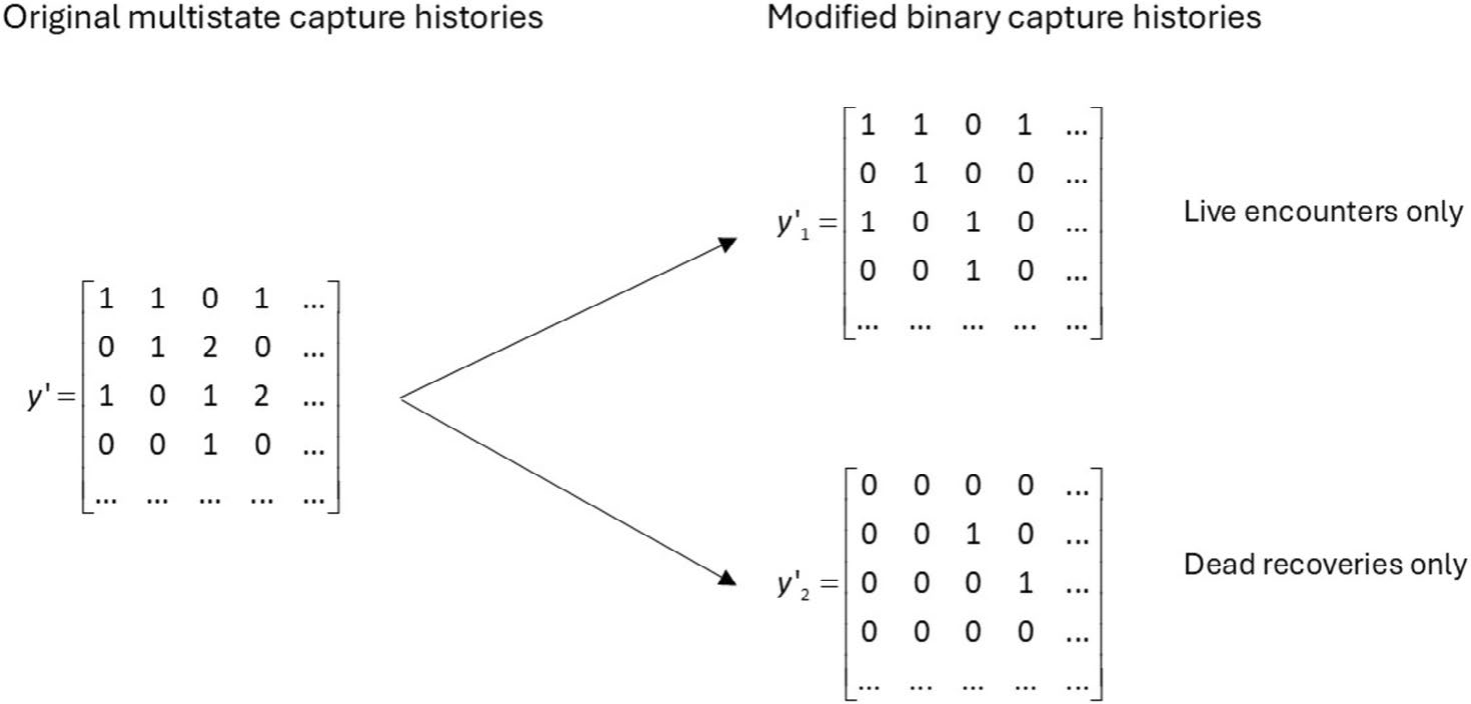
Illustration how the original multistate capture histories including live encounters (‘1’) and dead recoveries (‘2’) are transferred to binary capture histories which can be used for a single‐state state‐space model.

So far, the model is the same as the single‐state state‐space model using dead recoveries only (2.1.1.). However, for linking the latent state to our observations, we need a different model because we have two kinds of observations. As we want to model the observations with Bernoulli distributions, we have to transform our data into binary observations. We do that by splitting the recorded capture histories into two tables: the first contains information about the live encounters (***y'***_1_) and the second about the dead recoveries (***y'***_2_; see Figure 1). Then we use two Bernoulli trials to link the observed data with the latent states:

(10) $$\begin{array}{l} y{'}_{1,i,t}∼\text{Bernoulli}\left(z_{i,t}p\right)\\ y{'}_{2,i,t}∼\text{Bernoulli}\left(\left(z_{i,t-1}-z_{i,t}\right)r\right) \end{array}$$

#### 2.2.2 Multistate State‐Space Model

The original capture history data ***y′*** needs to be modified in order to be analyzed with the multistate model. The detection history now needs to distinguish between the observed states ‘encountered alive’ (1), ‘recovered dead’ (2) and ‘not encountered’ (3). Therefore, all 0's in the original ***y′*** are replaced by 3's, and we denoted these data ***v′***.

The model is very similar to the multistate state‐space model for the dead‐recovery data only (2.1.2). Although the definition of the latent states and the state‐transition matrix (Ω) is the same as shown in Equation (2), the observation process is slightly different, because living individuals can be encountered. It includes *p*, the probability to reencounter a living individual. Hence, the observation matrix is:

(11) $$\Theta =\left[\begin{array}{ccc} p & 0 & 1-p\\ 0 & r & 1-r\\ 0 & 0 & 1 \end{array}\right]$$

The implementation of this model as a state‐space model uses categorical distributions, and is the same as Equation (4). To distinguish this model from the following model, we name it ‘classical’ multistate state‐space model.

#### 2.2.3 Multistate State‐Space Model, BPA Variant

This is very similar to the corresponding model for the dead‐recovery data only (2.1.3). We again include the recovery of dead individuals in the state process which necessitates a different definition of the latent states: ‘alive’ (1), ‘recently dead and recovered’ (2) and ‘recently dead and not recovered, or long dead’ (3). The state‐transition matrix is defined exactly in the same way as for the dead‐recovery data only following Equation (5). To model the encounter of living individuals, the observation matrix is the following:

(12) $$\Theta =\left[\begin{array}{ccc} p & 0 & 1-p\\ 0 & 1 & 0\\ 0 & 0 & 1 \end{array}\right]$$

The implementation as a state‐space model is done using Equation (4), and the data are also the same (***v′***).

#### 2.2.4 Marginalized Single‐State State‐Space Model

The single‐state state‐space model of Section 2.2.1 is marginalized in a similar way to the dead‐recovery model (Section 2.1.4). The algorithm to compute the likelihood of each capture history requires information for each individual on occasions when it was known to be alive (between marking and the last live encounter) and when it was known to be dead (after a possible dead recovery). This information can be extracted from the data ***y′*** and must be used in addition to the data ***y′*** prepared as shown in Figure 1. We also applied this model to the pooled capture histories.

#### 2.2.5 Marginalized Multistate State‐Space Model

The multistate state‐space model of Section 2.2.2 is marginalized in a similar way as described for the dead‐recovery model (Section 2.1.5). The likelihood of the capture histories is calculated based on the state transition matrix (Equation (2)) and the observation matrix (Equation (11)). The data used is ***y′***. We also applied this model to the pooled capture histories.

#### 2.2.6 Multistate Multinomial Model

From the individual capture histories (***w*** ^***I***^: is the same as ***v′***, but all ‘3’ are replaced by ‘0’), the multistate m‐array is computed as described in section 2.1.7. These data are analyzed with a multistate multinomial model which is almost the same as the corresponding model for the dead‐recovery data only (2.1.7.). The only difference is the observation matrix, which now has to include the live encounters:

(13) $$\Gamma =\left[\begin{array}{ccc} p & 0 & 0\\ 0 & r & 0\\ 0 & 0 & 0 \end{array}\right]$$

From the state‐transition and observation matrices (Equations (2) and (13)), the cell probabilities of matrix $\tau_{t}$ needed for the multinomial likelihood to analyze the multistate m‐array data are calculated as described in section 2.1.7. The likelihood, finally, is computed as shown in Equation (9).

### 2.3 Data Simulation

We simulated dead‐recovery and joint dead‐recovery and live‐encounter data that were analyzed with the different models presented in sections 2.1 and 2.2. We first randomly simulated a value of survival from a uniform distribution ranging from 0.1 to 0.9, a value of recovery from a uniform distribution ranging from 0.05 to 0.4, and a value of encounter probability from a uniform distribution ranging from 0.1 to 0.9. These ranges were chosen to cover a wide range of life histories and sampling designs, but in a realistic way (hence the upper bound for dead recovery is 0.4). Once these values were chosen, capture histories were simulated using Bernoulli or categorical distributions (Kéry and Schaub 2012; Schaub and Kéry 2022). We assumed that there are 8 occasions (years) and that in each occasion, except the last, 100 individuals are newly marked and released. This results in a data set of capture histories for 700 individuals. In total, we generated 200 data sets. The R functions to simulate the data are provided in the vogelwarte.ch Open Repository and Archive https://doi.org/10.5281/zenodo.15340775 (Schaub and Badia‐Boher 2025).

The data were analyzed using the models presented above, implemented in the Bayesian framework. In all models, we used the uniform distribution in the range from 0 to 1 to specify vague priors for the model parameters (survival, recovery, re‐encounter).

The Bayesian framework uses Markov chain Monte Carlo (MCMC) simulation to draw samples from the posterior distribution, and initial values for the model parameters are needed to start the Markov chain. Usually, an initial value of a model parameter is generated from a draw of its prior distribution. Typically, multiple chains are used, starting from different values, and these chains must converge to a stable state. When the chains are run long enough and have reached a stable state, they are independent of the initial values. Therefore, the choice of initial values is a necessary step to ensure that the model runs but does not affect the outcome (the posterior distribution).

As latent state values are estimated in all non‐marginalized state‐space models, they require initial values for them in addition to the initial values for survival, recovery, and re‐encounter needed in all models considered here. Choosing initial values for latent states can be tricky because they need to be consistent with the model and the data. For individuals that are recovered dead, the latent states of the entire capture history are known. However, for individuals that are not recovered dead, the latent states are only known from marking until the last live encounter. After the last live encounter (or after marking if no live encounter occurs), the latent states are unknown. To initialize these unknown latent states, we have used three options: first, we assumed that all of these individuals survive until the end of the study; second, we assumed that they die immediately after the last encounter; and third, we assumed that the time of death is a random time between the last encounter and the end of the study.

For the dead‐recovery data, seven models were evaluated, and for those where the latent states had to be estimated, we used the three variants of initial values for the latent state variable described above. The marginalized models were fitted to the pooled capture histories in addition, resulting in a total of 15 analyses per simulated dataset. For the joint dead‐recovery and live‐encounter data, a total of six models were used, resulting in 14 analyses per data set due to the options regarding initial values.

All analyses were performed in R (version 4.2.2.; R Core Team 2018). The models were run with the R package NIMBLE (version 1.0.1; de Valpine et al. 2017). We always run 3 chains to check convergence based on the R‐hat (Brooks and Gelman 1998), but the burn‐in length and number of iterations differed between models, because they did not converge all at the same rate (Table 2). Following Turek et al. (2016) we calculated the computational efficiency of each model as the number of effective independent posterior samples obtained per second for the least converged parameter. For these calculations, all models were run with four MCMC chains for 30,000 iterations, of which the first 15,000 were discarded. We also fit the models using software JAGS (version 4.3.1; Plummer 2017) run from R package jagsUI (Kellner 2019) in order to check whether the findings were software‐specific and to compare computational efficiency.

**TABLE 2:** Overview of the different models and MCMC specifications used. Given are the model type, the sections where the model is described, the data types (D: Dead‐recovery data; LD: Joint dead‐recovery and live‐encounter data) and the MCMC specifications (burn‐in and number of iterations).

Type	Section	Data	Burn‐in	Iterations
Single‐state state‐space	2.1.1	D	2000	5000
Multistate state‐space, classical	2.1.2	D	2000	5000
Multistate state‐space, BPA version	2.1.3	D	2000	5000
Single‐state state‐space, marginalized	2.1.4	D	1000	3000
Multistate state‐space, marginalized	2.1.5	D	1000	3000
Single‐state multinomial	2.1.6	D	1000	3000
Multistate multinomial	2.1.7	D	1000	3000
Single‐state state‐space	2.2.1	LD	1000	3000
Multistate state‐space, classical	2.2.2	LD	2000	5000
Multistate state‐space, BPA version	2.2.3	LD	2000	5000
Single‐state state‐space, marginalized	2.2.4	LD	1000	3000
Multistate state‐space, marginalized	2.2.5	LD	1000	3000
Multistate multinomial	2.2.6	LD	1000	3000

To summarize the results, we first checked whether the MCMC chains had converged, which we defined as an R‐hat < 1.1 (Brooks and Gelman 1998). Simulated data producing estimates that have not converged were removed, and another data set was created and analyzed. This was repeated until 200 converged simulation runs were available. We then compared the posterior means of the target parameters with the data‐generating values. A strong correlation between them indicates that the model was able to recover the parameters, i.e., the model can be used for the estimation. We also computed the mean absolute bias (across simulations) of the estimated parameters and their coverage.

Code for all the simulation and analyses in R, NIMBLE, and JAGS are available on the vogelwarte.ch Open Repository and Archive https://doi.org/10.5281/zenodo.15340775 (Schaub and Badia‐Boher 2025).

## 3 Results

### 3.1 Dead‐Recovery Data Only

**FIGURE 2:**
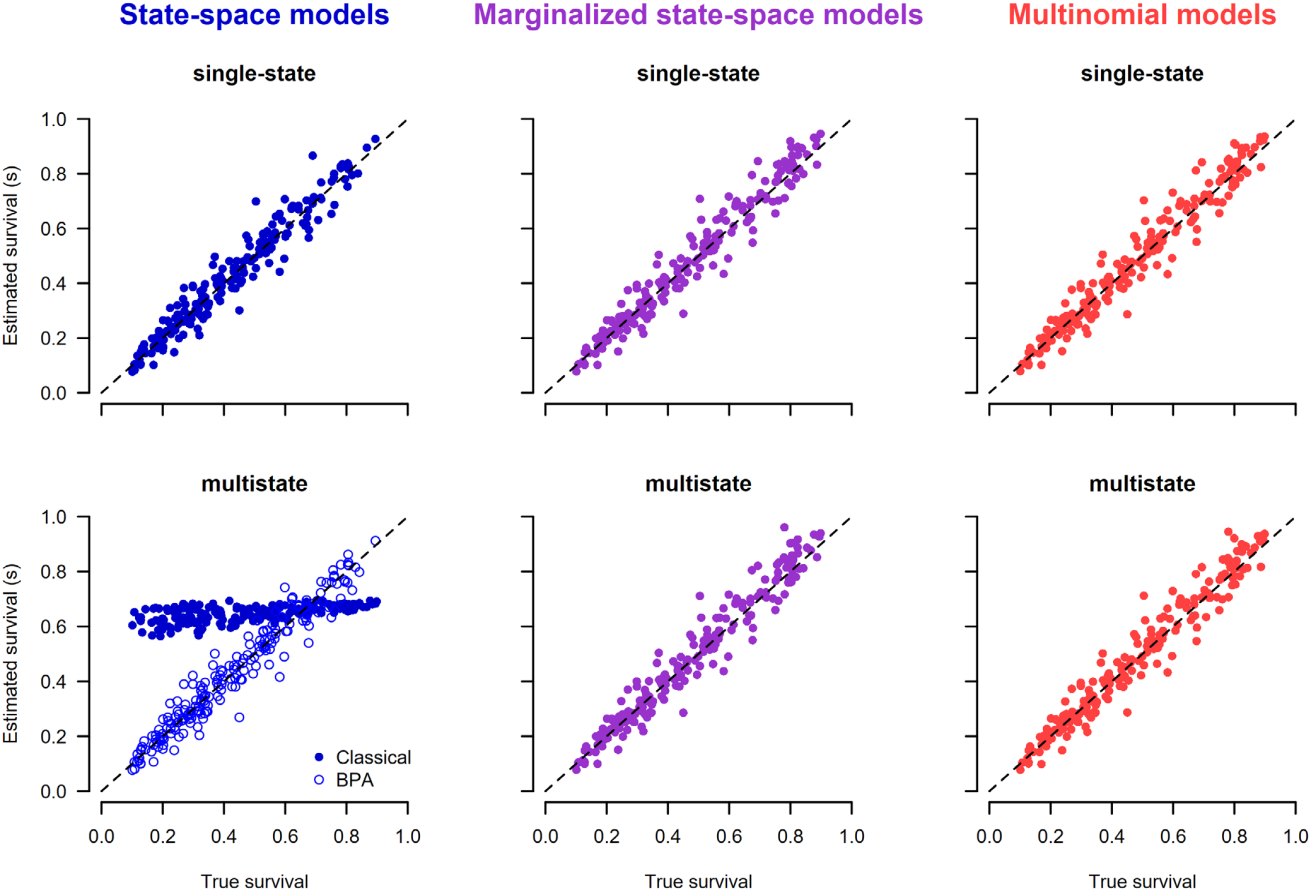
Scatterplots of posterior means versus the true values of survival obtained from different models analyzing dead‐recovery data. The initial values for the latent state of the state‐space models (left column) were generated assuming a random time of death. Each of the 200 converged simulation runs produced one point in the graphs.

**FIGURE 3:**
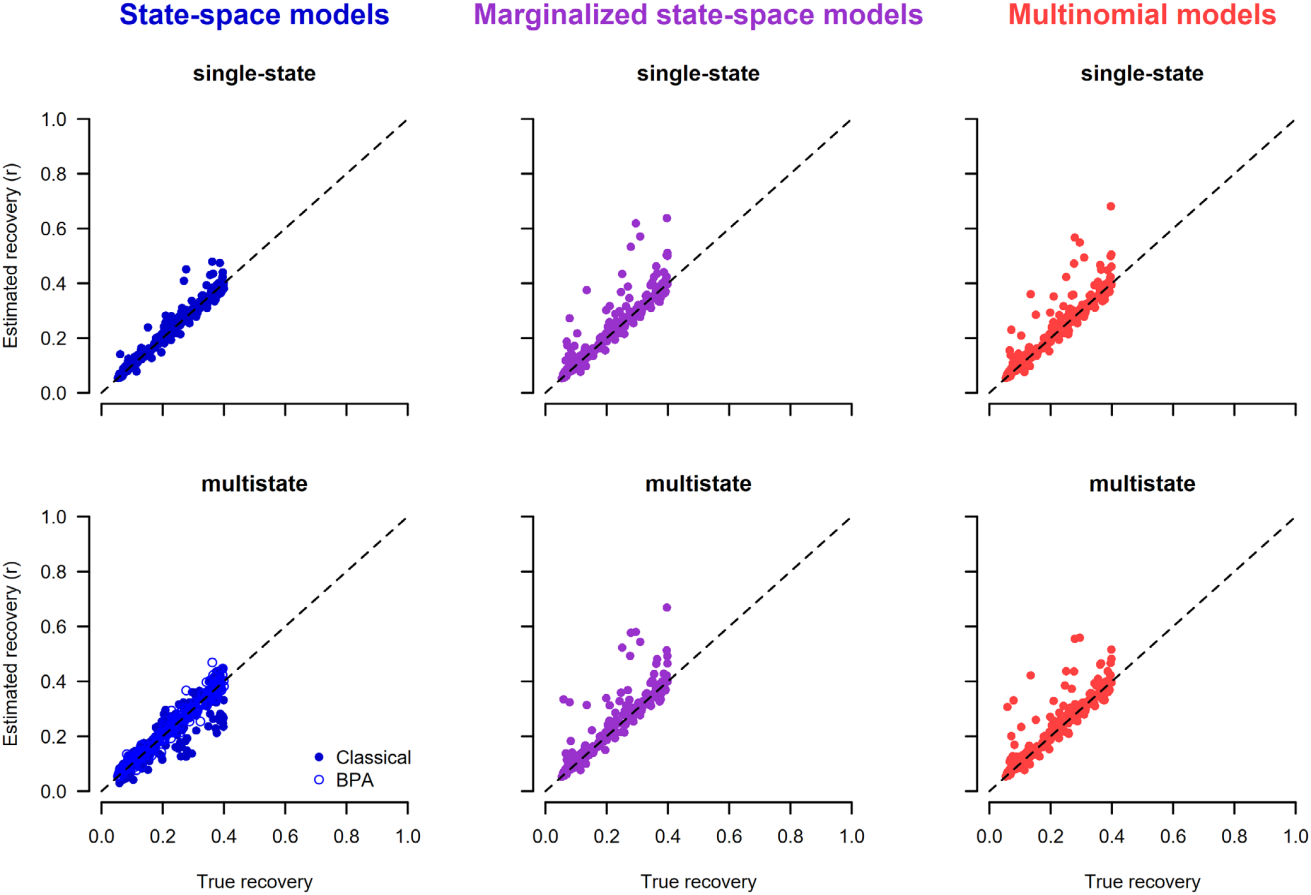
Scatterplots of posterior means versus the true values of recovery probabilities obtained from different models analyzing dead‐recovery data. The initial values for the latent state of the state‐space models (left column) were generated assuming a random time of death. Each of the 200 converged simulation runs produced one point in the graphs.

The posterior means of each simulation under each of the seven models are plotted against the data generating values (Figures 2 and 3). For all models except the classical multistate state‐space model (2.1.2), there was a clatter of points along the 1:1 line for the two target parameters. In particular, all models in which the latent states were not estimated, either because they were marginalized or because they used the multinomial likelihood, worked correctly. Of the state‐space models that estimated the latent states, the single‐state model and the BPA re‐parameterization of the multistate correctly recovered the data‐generating parameters. The only model that did not work was the classical multistate state‐space model. The choice of initial latent state values had no effect on the posteriors for the working state‐space models, whereas for the non‐working multistate state‐space model, the estimates depended on the chosen initial values (Figures S1 and S2). As expected, the estimates from the marginalized state‐space models were the same whether unpooled or pooled data were used (Figure S6).

The mean bias was usually small, and the coverage was close to the expected 0.95 for all models and initial values, except for the classical multistate state‐space model. For the latter, the bias was substantial, and the coverage was very poor, and the magnitude of the bias and coverage depended strongly on how the latent state variables were initialized.

In terms of computational efficiency, the single‐state multinomial and the marginalized multistate state‐space models using pooled data produced the most independent samples from the posterior distribution per unit time (Figure 7). The single‐state state‐space model and the BPA re‐parameterization of the multistate state‐space model were the least computationally efficient. The most efficient model (single‐state multinomial) was more than 6000 times more efficient than the least efficient model (BPA multistate state‐space).

### 3.2 Dead‐Recovery and Live‐Encounter Data

**FIGURE 4:**
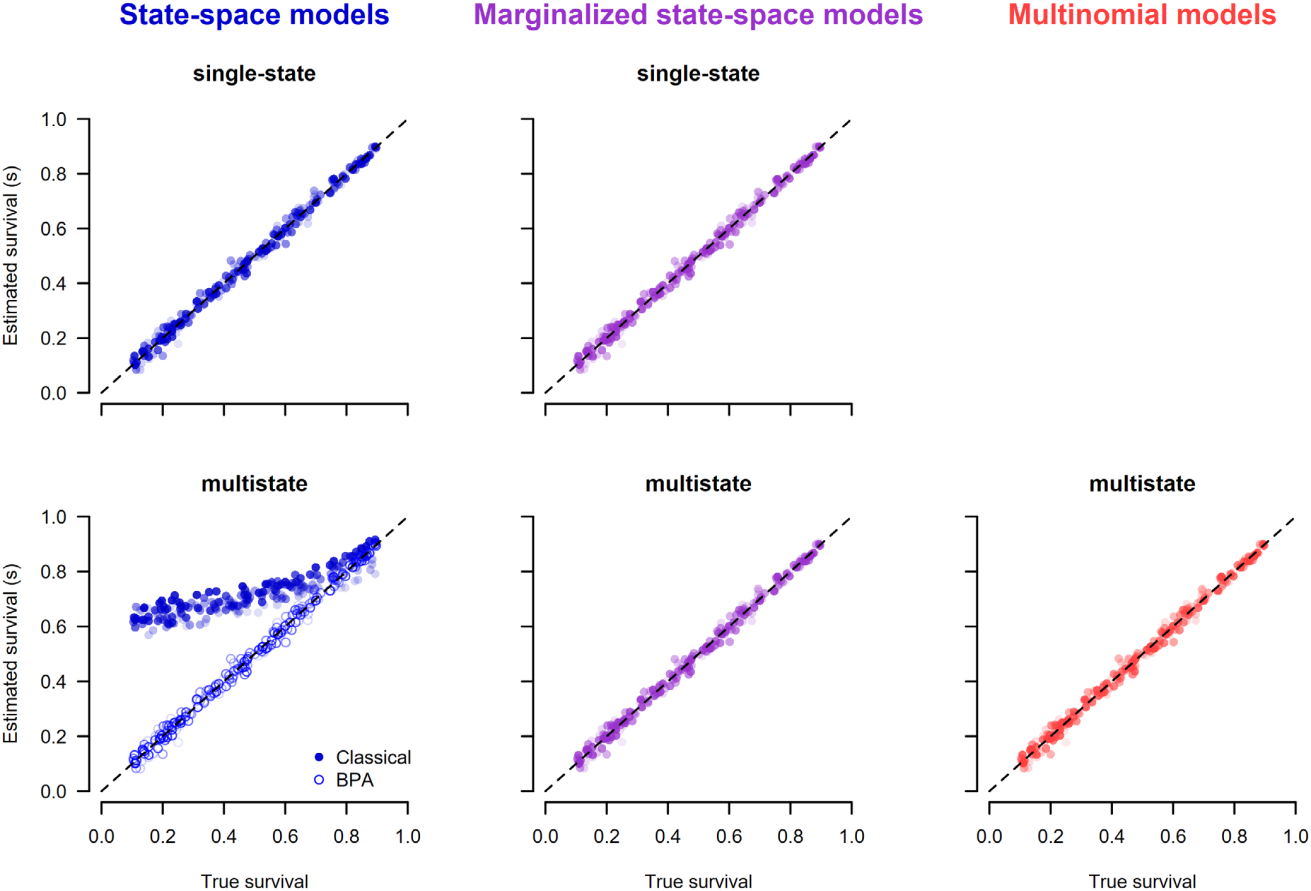
Scatterplots of posterior means versus the true values of survival obtained from different models jointly analyzing dead‐recovery and live‐encounter data. The initial values for the latent state of the state‐space models (left column) were generated assuming a random time of death. The color gradient shows the values of the recapture probabilities used to simulate the data (the darker, the higher the probability of recapture). Each of the 200 converged simulation runs produced one point in the graphs.

**FIGURE 5:**
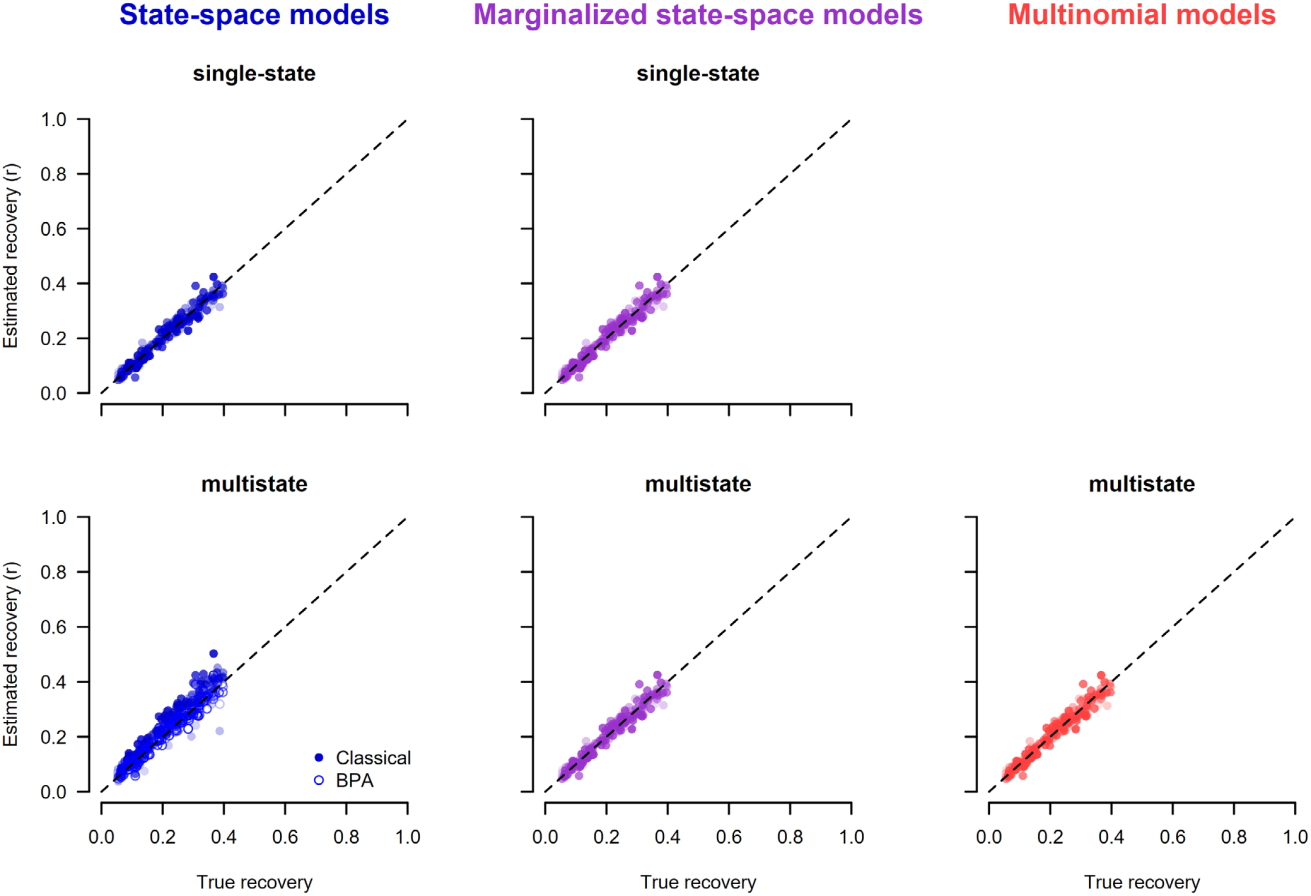
Scatterplots of posterior means versus the true values of recovery probabilities obtained from different models jointly analyzing dead‐recovery and live‐encounter data. The initial values for the latent state of the state‐space models (left column) were generated assuming a random time of death. The color gradient shows the values of the recapture probabilities used to simulate the data (the darker, the higher the probability of recapture). Each of the 200 converged simulation runs produced one point in the graphs.

**FIGURE 6:**
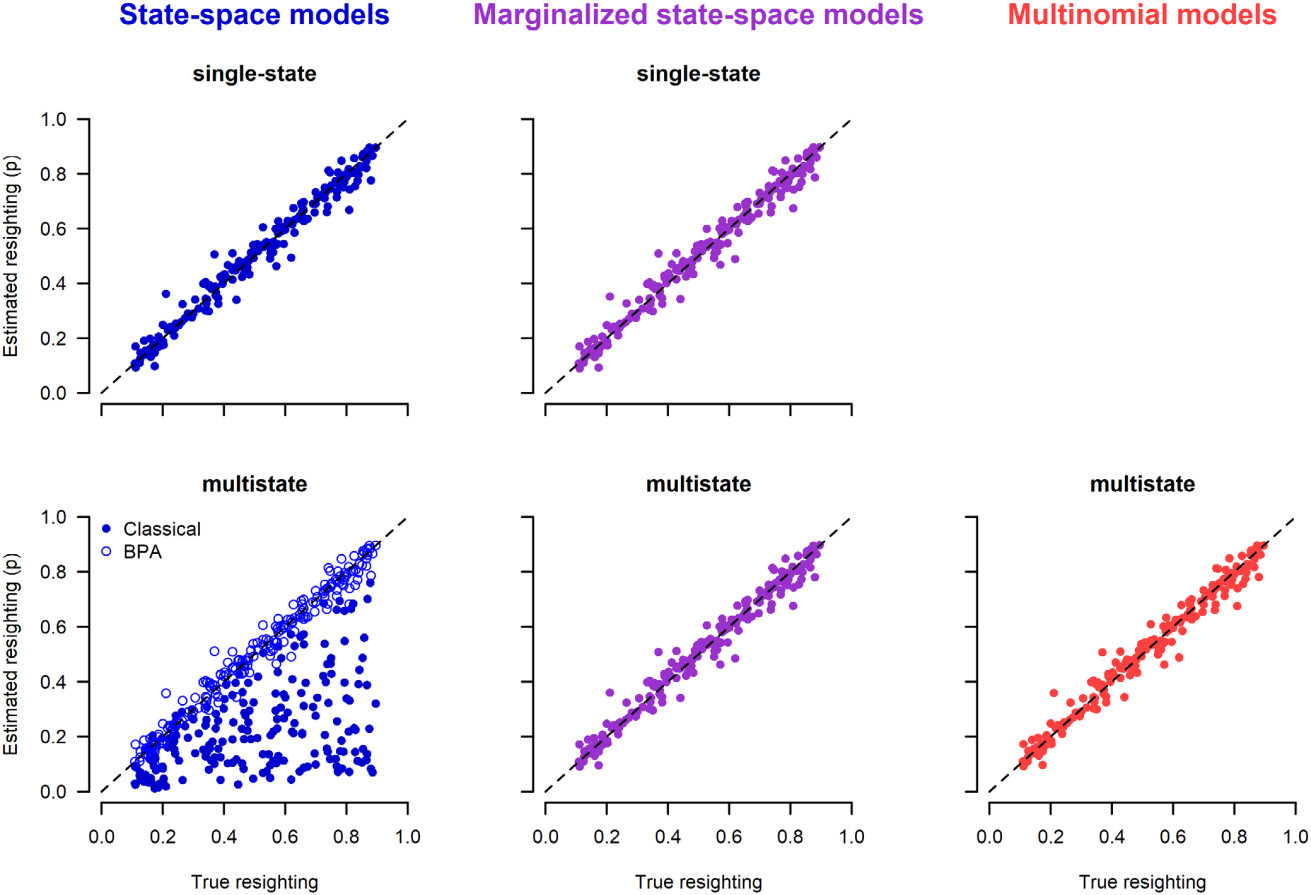
Scatterplots of posterior means versus the true values of resighting probabilities obtained from different models jointly analyzing dead‐recovery and live‐encounter data. The initial values for the latent state of the state‐space models (left column) were generated assuming a random time of death. Each of the 200 converged simulation runs produced one point in the graphs.

The posterior means of each parameter and simulation under each of the six models are shown in Figures 4, 5, 6. The general results are the same as for the models that use dead‐recovery data only: with the exception of the classical multistate state‐space model (2.2.2), all models produced parameter estimates that matched the data‐generating values, were unbiased, and had appropriate coverage (Table 3). In contrast, the classical multistate state‐space model produced parameter values that were biased and had poor coverage (Table 3). Furthermore, the estimates from this model were dependent on the chosen initial values of the latent states (Figures S3–S5), whereas there was no dependence on the initial values for the single‐state and BPA multistate state‐space models. As expected, the estimates from the marginalized state‐space models were the same whether unpooled or pooled data were used (Figure S6). Estimates of survival and recovery appeared to be slightly closer to the data‐generated values in the joint model with live encounters (Figures 4 and 5) than in the dead‐recovery only model (Figures 2 and 3). This is because there was more information on survival in the joint model due to the live‐encounter data.

**TABLE 3:** Mean absolute bias and coverage (probability that the 95% credible interval includes the true parameter value) of the target parameters for the different models based on 200 simulations. SS: State‐space model. marg.: Marginalized. D: Dead‐recovery data. LD: Joint dead‐recovery and live‐encounter data.

Model	Initials for ***z***	Data	Survival		Recovery		Reencounter	
			Bias	Coverage	Bias	Coverage	Bias	Coverage
Single‐state SS	Alive	D	0.005	0.935	0.001	0.965	—	—
	Dead	D	0.006	0.935	0.002	0.945	—	—
	Random	D	0.006	0.960	0.004	0.940	—	—
Single—state SS, marg.	—	D	0.019	0.955	0.008	0.935	—	—
Single—state SS, marg.	—	Pooled D	0.018	0.960	0.007	0.940	—	—
Multistate SS, classical	Alive	D	0.759	0.000	0.456	0.000	—	—
	Dead	D	−0.041	0.000	−0.373	0.545	—	—
	Random	D	−0.004	0.055	0.158	0.585	—	—
Multistate SS, BPA	Alive	D	0.006	0.925	0.002	0.945	—	—
	Dead	D	0.005	0.935	0.003	0.950	—	—
	Random	D	0.003	0.930	0.001	0.935	—	—
Multistate SS, marg.	—	D	0.021	0.930	0.009	0.955	—	—
Multistate SS, marg.	—	Pooled D	0.022	0.945	0.009	0.935	—	—
Single‐state multinomial	—	D	0.018	0.945	0.007	0.955	—	—
Multistate multinomial	—	D	0.018	0.940	0.007	0.950	—	—
Single‐state SS	Alive	LD	−0.001	0.945	0.000	0.925	−0.001	0.940
	Dead	LD	−0.001	0.945	0.000	0.945	−0.001	0.925
	Random	LD	−0.001	0.940	−0.001	0.935	−0.001	0.940
Single‐state SS, marg.	—	LD	−0.001	0.945	0.000	0.935	−0.001	0.945
Single‐state SS, marg.	—	Pooled LD	−0.001	0.940	0.000	0.935	−0.001	0.945
Multistate SS, classical	Alive	LD	0.468	0.000	0.770	0.000	−0.365	0.000
	Dead	LD	−0.096	0.140	−0.017	0.765	0.181	0.015
	Random	LD	0.233	0.055	0.026	0.590	−0.275	0.030
Multistate SS, BPA	Alive	LD	−0.001	0.945	−0.001	0.930	−0.001	0.950
	Dead	LD	−0.001	0.950	0.000	0.925	−0.001	0.945
	Random	LD	−0.001	0.940	0.000	0.930	−0.001	0.955
Multistate SS, marg.	—	LD	−0.001	0.950	0.000	0.935	−0.001	0.940
Multistate SS, marg.	—	Pooled LD	−0.001	0.945	0.000	0.920	−0.001	0.950
Multistate multinomial	—	LD	−0.001	0.945	0.000	0.940	−0.001	0.935

**FIGURE 7:**
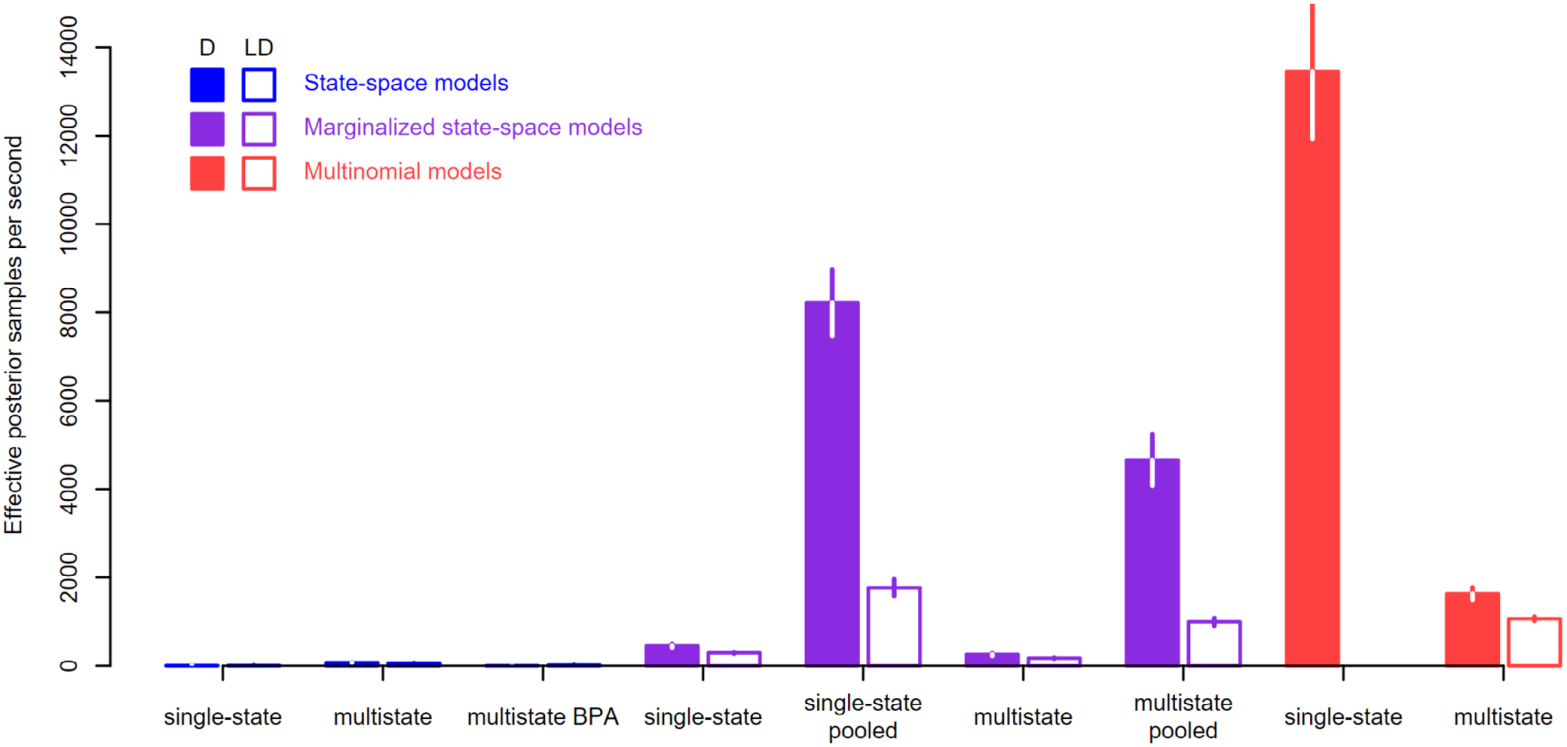
Computational efficiency of the different models measured as the number of independent effective posterior samples per second (compilation time not included). The filled bars show the mean efficiency (across 10 data sets) of the dead‐recovery models (D), the open bars show the mean efficiency of the models for the joint analysis of dead‐recovery and live‐encounter data (LD). The vertical lines show mean ± 1SD of efficiency. Note that the first three models on the left are state‐space models (color hard to see).

In terms of computational efficiency, the marginalized single‐state state‐space model with pooled data produced the most independent samples from the posterior distribution per unit time, closely followed by the marginalized multistate model with pooled data and the multistate multinomial model (Figure 7). The single‐state and BPA multistate state‐space models were less efficient, and the ratio between the most efficient (marginalized single‐state with pooled data) and the least efficient model (BPA multistate) was about 300.

The results obtained showed the same patterns when JAGS is used instead of NIMBLE (Figures S7–S9, Table S1) and were therefore not software specific.

## 4 Discussion

We present several models that can be used to analyze dead‐recovery data alone, or jointly with live‐encounter data, in a Bayesian framework to estimate survival and detection parameters. These include single‐ and multistate state‐space models, marginalized single‐ and multistate state‐space models, and single‐ and multistate multinomial likelihoods. We demonstrate that the classical multistate state‐space model, which works well with frequentist methods (Lebreton et al. 1999), yields biased parameter estimates in the Bayesian framework when using standard software such as NIMBLE or JAGS, confirming the findings of Kéry and Schaub (2012) for WinBUGS. That this model produces biased estimates is not well known in the literature (but see Kéry and Schaub 2012) and may be a source of error. We have shown that multiple alternative models are available that produce unbiased parameter estimates, allow the same modeling flexibility as the classical multistate state‐space model, and are often computationally more efficient (see discussion below).

It seems paradoxical that the classical multistate state‐space model does not work when dead recoveries are included in the data. The model is mathematically correct and works well in the frequentist framework where it has successfully been applied in several studies (e.g., Duriez et al. 2009; Péron et al. 2013; Folliot et al. 2020). So why does the model not work when the Bayesian framework is used? The problem is the MCMC sampling algorithm implemented in BUGS software such as NIMBLE, JAGS, or WinBUGS (R.Pradel and F. Ketwaroo, pers. comm.). The algorithm is a first‐order Markov chain, but in the classical multistate state‐space model with dead recoveries, there are deterministic transitions over two time steps. If an individual has died between *t* and *t* + 1, it transitions from state 1 (alive) at *t* to state 2 (recently dead) at *t* + 1, and from there to state 3 (long dead) at *t* + 2 with probability 1. This causes the standard samplers to not update correctly. Even after the model has converged, the estimated parameters depend on how the initial values for the latent states are generated. This is a clear indication that the samplers do not work properly. For NIMBLE, it is possible to write a custom MCMC algorithm that samples nodes of latent states jointly rather than independently to overcome this problem (R. Pradel, pers. comm.; see also Pradel 2021). Because alternative models are available, we have not followed the path of writing a customized sampler, which is a challenging task for ecologists.

We have shown that there are several alternative models that can be used to estimate survival from dead‐recovery data alone or in combination with live encounters. These models all produce unbiased parameter estimates, so the question is which one to use. The models differ in their computational efficiency and in their ability to model the desired effects, and we believe that these two qualities should be considered when choosing a model.

Computation was generally much more efficient when using either the multinomial likelihood or the marginalized state‐space model with pooled capture histories than when using unmarginalized state‐space models (Figure 7). The computational efficiency of the models shown is specific to the size of the data, which in our case was 700 individuals on 8 occasions. If there were more individuals, we would expect computational efficiency to remain the same for the multinomial model, and to decrease for all other models, although probably at different rates. If there were more occasions, the computational efficiency would decrease in all models, but again perhaps not by the same amount. The reparametrized multistate state‐space model (BPA variant) was particularly inefficient and is therefore not recommended for large datasets, as it could easily lead to intractable computational times.

The different models shown were simple because the model parameters were constant over time and had no individual effects. It is straightforward to make these models more complex and to include effects that the researcher is interested in or that are necessary to account for heterogeneity or overdispersion. However, it is not possible in all models to include all effects in an efficient way. Modeling temporal effects, with fixed or random time effects, or as a function of temporal covariates, on survival and detection parameters can be implemented similarly in all models (Table 4). It is also possible to include discrete individual effects that do not change over time such as gender or color morph in all models. In the state‐space models, this would require the inclusion of an individual covariate including group membership. For the multinomial model, this would require either splitting the data by group (e.g., producing an m‐array for each group) and adapting the model accordingly or creating new states to indicate group membership. For the marginalized state‐space models using pooled data, this would require considering not only unique capture histories but also group membership when pooling the data. Computational efficiency is not expected to change if group membership can be modeled as a single covariate but will decrease if the data have to be split or pooled into different groups. The inclusion of continuous individual effects is only possible when using state‐space models and marginalized state‐space models with unpooled data (Table 4) but not in models where individual capture histories are summarized (in a m‐array or pooled). In this scenario, the most computationally efficient models are likely to be the marginalized models applied to unpooled data. Additional states such as geographical locations or causes of mortality (Schaub and Pradel 2004) can be included in all multistate models but not in single‐state models. Age effects (or similar transients, Pradel et al. 1997) can be included in all models but not always in the same way. In all multistate models, age information can be included as additional states, and in the state‐space and marginalized models, age information can be included as a time‐varying individual discrete covariate (Kéry and Schaub 2012). The computational time is not expected to change significantly when age information is included as a covariate but is expected to increase notably with an increasing number of states. In multinomial models, individual identity is lost, which prevents the inclusion of age information as a covariate. Instead, age‐dependent m‐arrays can be used. This requires that the cell probabilities include age‐dependent parameters (Schaub and Kéry 2022), which is all feasible but more complex to code. Therefore, the simplest and most common way to account for age in multinomial models is to add age classes as states. This leads to an increase in the dimensionality of the m‐array, which can significantly reduce computation efficiency. A similar consideration is the inclusion of an immediate trap response (Pradel 1993): again, it can be included by using additional states or by an individual discrete covariate (Kéry and Schaub 2012).

**TABLE 4:** Overview of whether and how effects can be implemented in the different models. For continuous individual effects and more states (e.g., geographical locations) the table shows whether the implementation is possible (X) or impossible (—). For modeling age, transients, and immediate trap responses, the table shows how implementation is possible, either by a time‐varying, individual discrete covariate (covariate), by extending the model with additional states (state), or using multiple m‐arrays. Note that temporal (e.g., annual variation) and discrete individual effects (e.g., sex) can be implemented in all models. They are therefore not shown explicitly in the table.

Model	Effects			
	Continuous individual	More states	Age/transients	Immediate trap response
Single‐state state‐space	X	—	Covariate	Covariate
Single‐state state‐space, marginalized	X	—	Covariate	Covariate
Single‐state state‐space, marginalized pooled data	—	—	Covariate	Covariate
Multistate state‐space, BPA	X	X	State or covariate	State or covariate
Multistate state‐space, marginalized	X	X	State or covariate	State or covariate
Multistate state‐space, marginalized pooled data	—	X	State or covariate	State or covariate
Single‐state multinomial	—	—	Multiple m‐arrays	Multiple m‐arrays
Multistate multinomial	—	X	State	State

We have shown that the joint analysis of dead recovery and live‐encounter data is possible with single‐state state space models, if the data are prepared accordingly (Figure 1). The marginalized version of this model was very efficient in terms of computational time, in particular when pooled data were used. This approach is attractive because it can include other types of observations (e.g., genetic encounters (Schaub et al. 2024) or the robust design information (Riecke et al. 2018)) and offers a lot of flexibility. For example, Schaub et al. (2024) used this model to estimate survival from dead recoveries and live encounters through resighting and genetic identification. During an occasion, a live individual may be encountered only through resighting, only through genetic identification, or both. This is easily modeled with this formulation, whereas classical multistate models had to increase the set of observed states, which would negatively affect the computational time. Furthermore, the simultaneous inclusion of age effects on resighting and immediate trap response can all be done using covariates and does not require additional states. In addition, it was the fastest of all models estimating latent states and may therefore be the best option when latent state estimates are of particular interest. We therefore believe that this model is attractive and should be in the modeler's toolbox, but has not yet seen many applications (Riecke et al. 2021; Schaub et al. 2024).

Model checking is an important step in any data analysis. The subject of goodness‐of‐fit (GoF) testing is considerably more advanced in frequentist than in Bayesian model formulations. As a result, frequentist GoF tests are often applied to data used in Bayesian models to provide diagnoses of fit. Among the most important frequentist GoF tests, Brownie et al. (1985) developed specific tests for dead‐recovery data under the multinomial model, which essentially compare observed data with expected values. Barker (1997) and McCrea et al. (2014) developed tests for the joint dead‐recovery and live‐encounter model, some of which are diagnostic of the possible cause of the lack of fit. In the Bayesian framework, posterior predictive checks could be applied to the corresponding test statistics (Conn et al. 2018). However, these tests are not specific to most of the Bayesian models presented here, but could be the subject of future research.

In summary, we have shown that there are various models with which dead‐recovery data, either alone or jointly with live‐encounter data, can be analyzed. It is important to realize that the classical multistate state‐space model, as formulated in Lebreton et al. (1999), works perfectly with frequentist methods but does not work in the Bayesian framework when standard MCMC samplers are used. Yet, there are alternatives that can be applied. The state‐space formulation is the approach that offers the greatest deal of modeling flexibility, as it allows a convenient inclusion of discrete and continuous individual covariates. The remaining models may be more restrictive in terms of modeling but are often more computationally efficient. Although multinomial models may excel at analyzing datasets with large numbers of individuals, the marginalized likelihood applied to pooled data may be the most efficient model structure for data that include large numbers of individual covariates (e.g., multiple age classes, combinations of age classes and groups). In cases where the user is in doubt as to which likelihood might be faster for their data, evaluating the efficiency of different likelihoods is a reasonable option.

## Author Contributions

**Michael Schaub:** conceptualization (equal), formal analysis (equal), writing – original draft (lead). **Jaume A. Badia‐Boher:** conceptualization (equal), formal analysis (equal), writing – review and editing (supporting).

## Conflicts of Interest

The authors declare no conflicts of interest.

## Supporting information


Appendix S1.

## Data Availability

There is no data in this manuscript. Code for data simulation and all analyses in R, NIMBLE, and JAGS are published on the vogelwarte.ch Open Repository and Archive https://doi.org/10.5281/zenodo.15340775 (Schaub and Badia‐Boher 2025).
