# Supplementary material for: Comparison of Bayesian Models to Estimate Survival From Dead‐Recovery Alone and Together With Live‐Encounter Data: Challenges and Opportunities
Source: Ecol Evol. 2025 Jun 1;15(6):e71517. doi: 10.1002/ece3.71517 (PMC12127138; doi:10.1002/ece3.71517)
Supplement: Supplementary file 1 — Appendix S1. [file ECE3-15-e71517-s001.pdf]

SUPPORTING INFORMATION

Comparison of Bayesian models to estimate survival from dead-recovery alone and together with live-encounter data: challenges and opportunities

Michael Schaub, Jaume A. Badia-Boher

Schweizerische Vogelwarte, 6204 Sempach, Switzerland

Corresponding author:

Michael Schaub, michael.schaub@vogelwarte.ch

Appendix 1: Additional figures

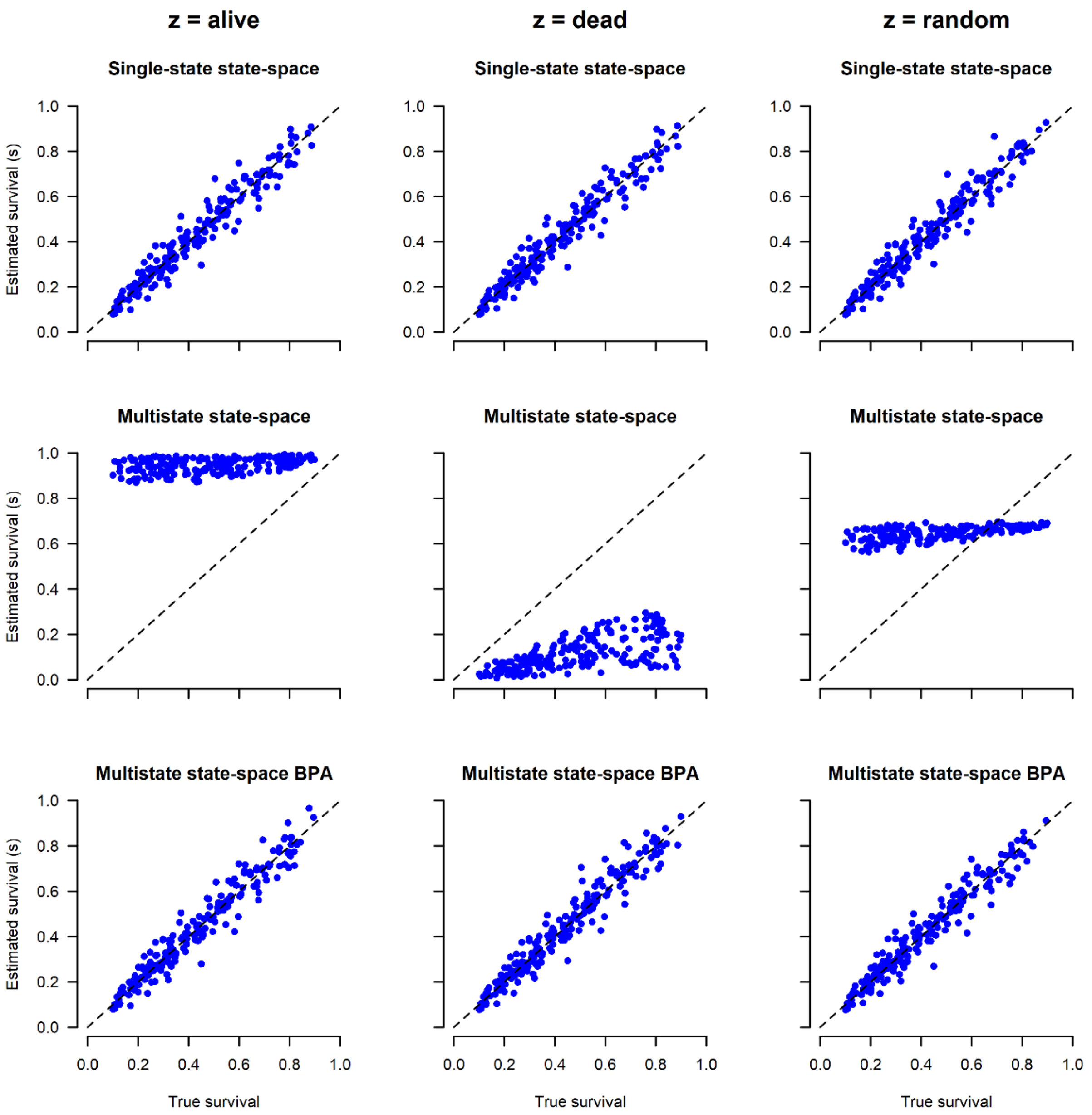

FIGURE S1. Scatterplots of posterior means versus the true values of survival obtained from different state-space models using different initial values for the latent state variable analyzing dead-recovery data. The initial values for the latent state assumed that all individuals were alive until the end of the study period (z = alive, left column), that all died immediately after the last encounter (z = dead, middle column), and that the time of death was randomly generated (z = random, right column). The panels in the last column correspond to the panels shown in Figure 2. Each of the 200 converged simulation runs produced one point in the graphs.

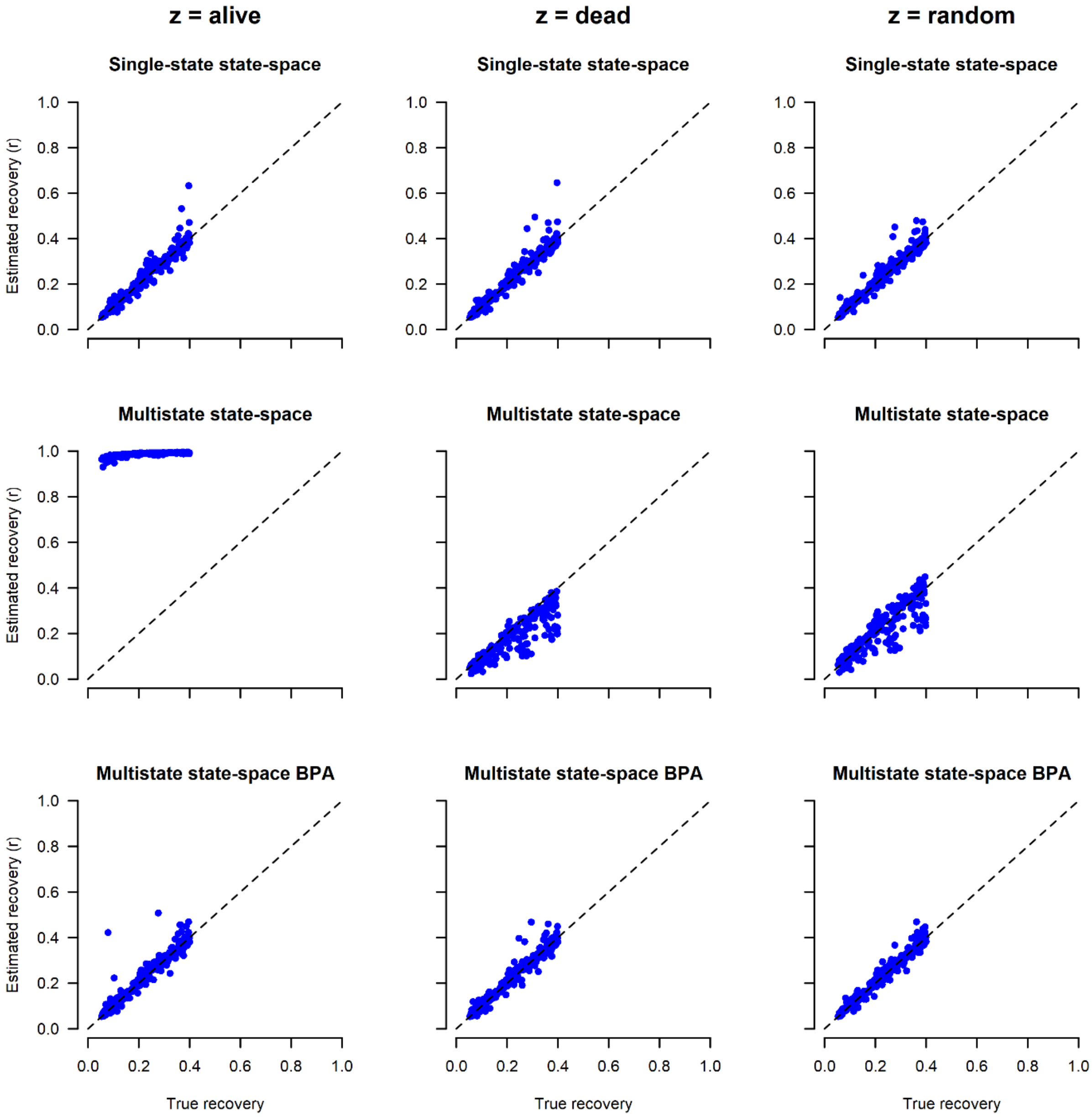

FIGURE S2. Scatterplots of posterior means versus the true values of recovery probabilities obtained from different state-space models using different initial values for the latent state variable analyzing dead-recovery data. The initial values for the latent state assumed that all individuals were alive until the end of the study period (z = alive, left column), that all died immediately after the last encounter (z = dead, middle column), and that the time of death was randomly generated (z = random, right column). The panels in the last column correspond to the panels shown in Figure 3. Each of the 200 converged simulation runs produced one point in the graphs.

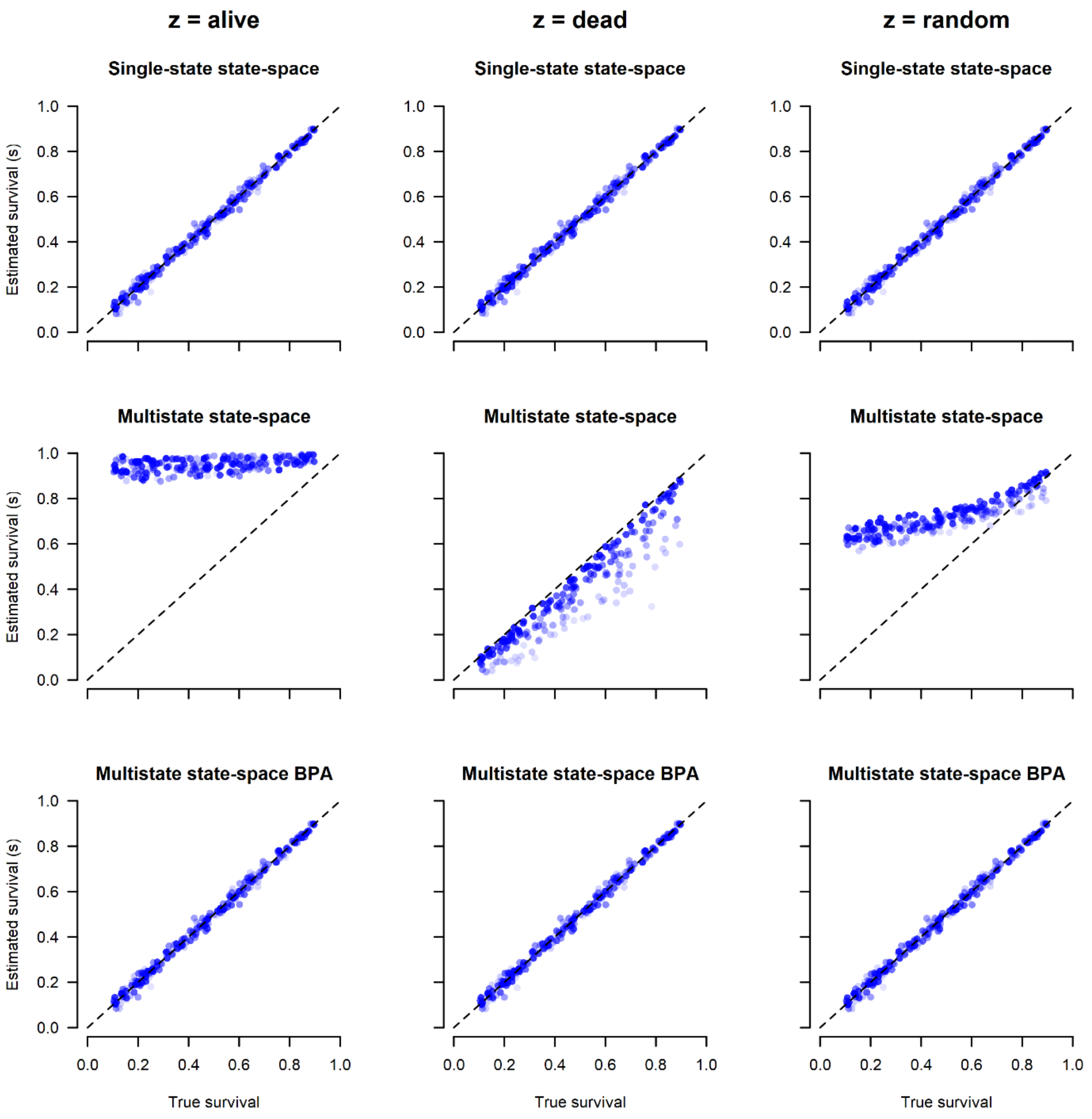

FIGURE S3. Scatterplots of posterior means versus the true values of survival obtained from different state-space models using different initial values for the latent state variable jointly analyzing dead-recovery and live-encounter data. The initial values for the latent state assumed that all individuals were alive until the end of the study period (z = alive, left column), that all died immediately after the last encounter (z = dead, middle column), and that the time of death was randomly generated (z = random, right column). The color gradient shows the values of the recapture probabilities used to simulate the data (the darker, the higher the probability of recapture). The panels in the last column correspond to the panels shown in Figure 4. Each of the 200 converged simulation runs produced one point in the graphs.

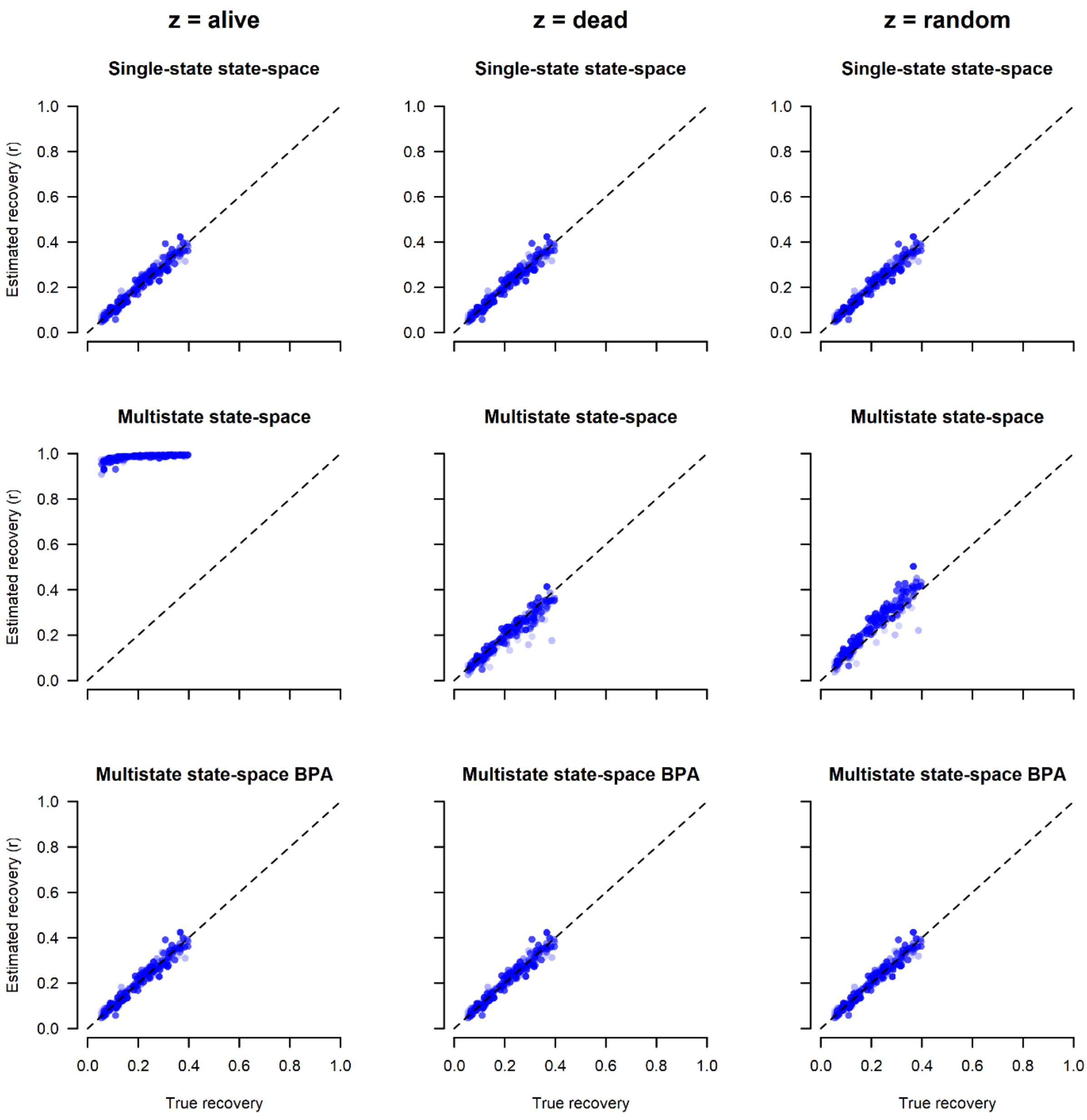

FIGURE S4. Scatterplots of posterior means versus the true values of recovery probabilities obtained from different state-space models using different initial values for the latent state variable jointly analyzing dead-recovery and live-encounter data. The initial values for the latent state assumed that all individuals were alive until the end of the study period (z = alive, left column), that all died immediately after the last encounter (z = dead, middle column), and that the time of death was randomly generated (z = random, right column). The color gradient shows the values of the recapture probabilities used to simulate the data (the darker, the higher the probability of recapture). The panels in the last column correspond to the panels shown in Figure 5. Each of the 200 converged simulation runs produced one point in the graphs.

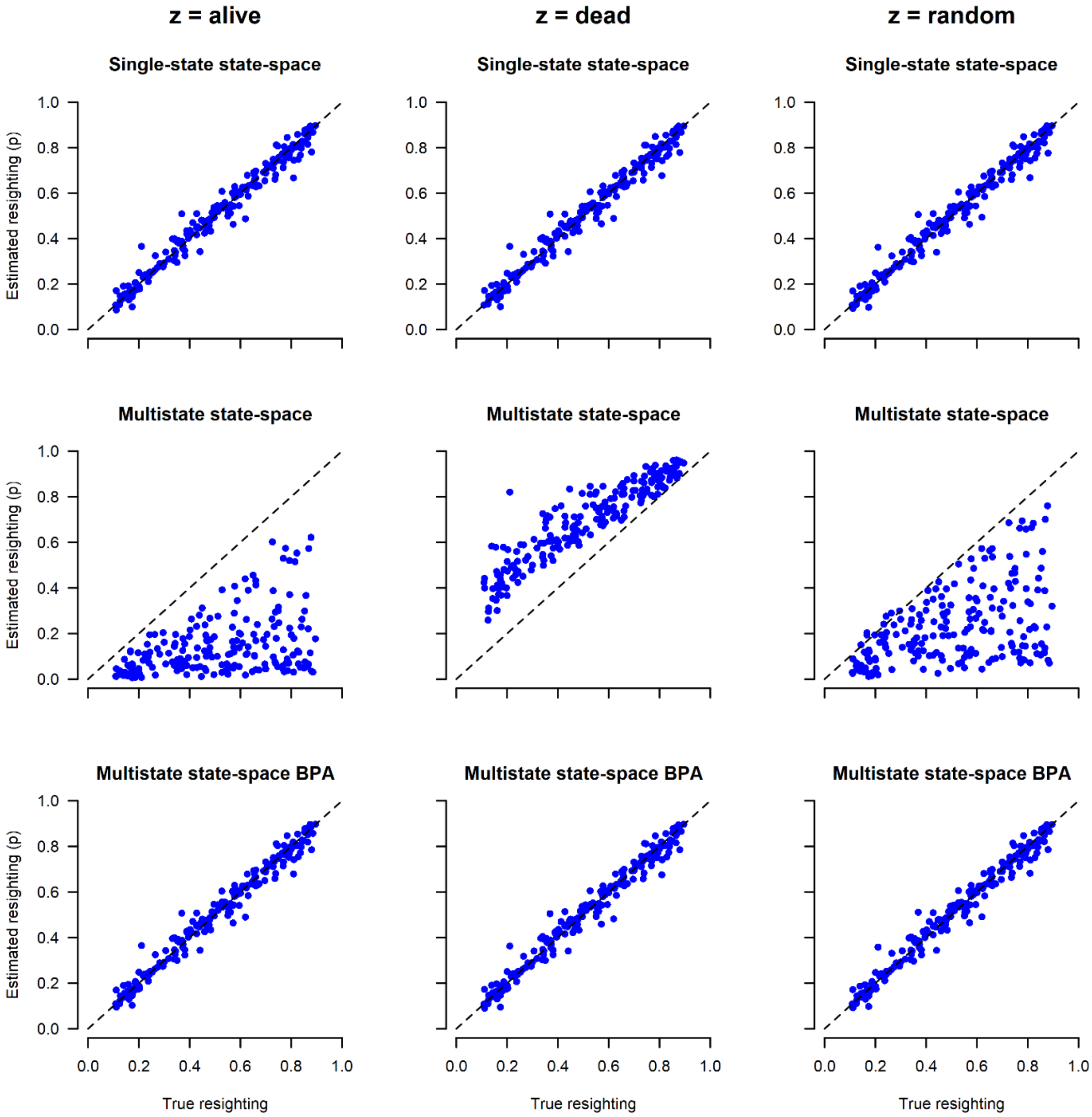

FIGURE S5. Scatterplots of posterior means versus the true values of resighting probabilities obtained from different state-space models using different initial values for the latent state variable jointly analyzing dead-recovery and live-encounter data. The initial values for the latent state assumed that all individuals were alive until the end of the study period (z = alive, left column), that all died immediately after the last encounter (z = dead, middle column), and that the time of death was randomly generated (z = random, right column). The panels in the last column correspond to the panels shown in Figure 6. Each of the 200 converged simulation runs produced one point in the graphs.

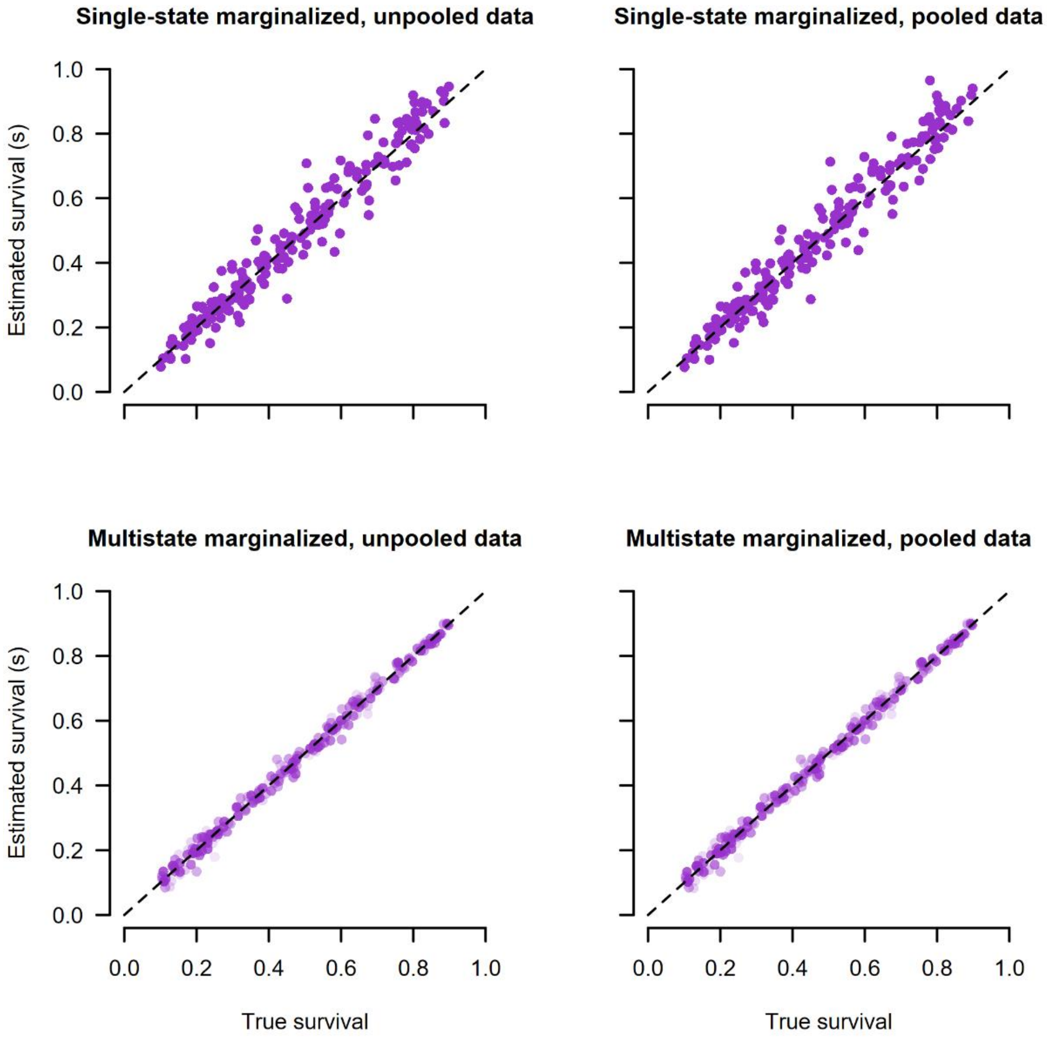

FIGURE S6. Scatterplots of posterior means versus the true values of survival probabilities obtained from marginalized state-space models. The panels at the top show the results from single-state models fitted to unpooled (left column) and pooled (right column) dead-recovery data. The bottom panels show the results from multistate models fitted to unpooled (left column) and pooled (right column) dead-recovery and live-encounter data. Each of the 200 converged simulation runs produced one point in the graphs.

Appendix 2: Results from JAGS

Data sets simulated with the same data generating parameters and using the same models as for NIMBLE were also analysed with JAGS. Here we present the table (Table S1) showing the bias and coverage of the parameter estimates (analogous to Table 3), as well as figures showing the posterior means against the data generating parameters (but restricted to survival; Figures S7 and S8), and a figure showing the computational efficiency (Figure S9).

TABLE S1. Mean absolute bias and coverage (probability that the 95% credible interval includes the true parameter value) of the target parameters for the different models based on 200 simulations. SS: state-space model. marg.: marginalized. D: dead-recovery data. LD: joint dead-recovery and live-encounter data.

			Survival		Recovery		Reencounter	
Model	Initials for ***z***	Data	Bias	Coverage	Bias	Coverage	Bias	Coverage
Single-state SS	Alive	D	0.000	0.940	0.001	0.950	-	-
	Dead	D	-0.001	0.950	0.000	0.950	-	-
	Random	D	0.000	0.960	0.000	0.965	-	-
Single-state SS, marg.	-	D	0.013	0.940	0.020	0.925	-	-
Single-state SS, marg.	-	Pooled D	0.011	0.955	0.020	0.935	-	-
Multistate SS, classical	Alive	D	0.428	0.000	0.757	0.000	-	-
	Dead	D	-0.400	0.000	-0.044	0.480	-	-
	Random	D	0.139	0.076	-0.013	0.576	-	-
Multistate SS, BPA	Alive	D	0.000	0.950	0.006	0.960	-	-
	Dead	D	0.001	0.955	0.007	0.960	-	-
	Random	D	0.000	0.955	0.009	0.945	-	-
Multistate SS, marg.	-	D	-0.003	0.950	0.008	0.945	-	-
Multistate SS, marg.	-	Pooled D	0.022	0.945	0.009	0.935	-	-
Single-state multinomial	-	D	0.002	0.950	0.013	0.965	-	-
Multistate multinomial	-	D	-0.007	0.950	0.002	0.950	-	-
Single-state SS	Alive	LD	0.001	0.955	0.003	0.930	0.001	0.935
	Dead	LD	0.001	0.965	0.002	0.950	0.004	0.935
	Random	LD	0.000	0.965	0.002	0.960	0.004	0.930
Single-state SS, marg.	-	LD	-0.001	0.960	0.003	0.970	0.002	0.980
Single-state SS, marg.	-	Pooled LD	-0.001	0.955	0.003	0.980	0.002	0.975
Multistate SS, classical	Alive	LD	0.429	0.000	0.757	0.000	-0.338	0.000
	Dead	LD	-0.097	0.120	-0.020	0.755	0.174	0.010
	Random	LD	0.172	0.084	0.016	0.639	-0.197	0.108
Multistate SS, BPA	Alive	LD	0.001	0.960	0.000	0.945	0.000	0.935
	Dead	LD	0.001	0.965	0.000	0.960	0.000	0.925
	Random	LD	0.001	0.960	0.001	0.960	-0.001	0.925
Multistate SS, marg.	-	LD	0.001	0.945	0.003	0.945	0.001	0.920
Multistate SS, marg.	-	Pooled LD	0.001	0.945	0.004	0.940	0.003	0.970
Multistate multinomial	-	LD	0.001	0.955	0.003	0.945	0.001	0.935

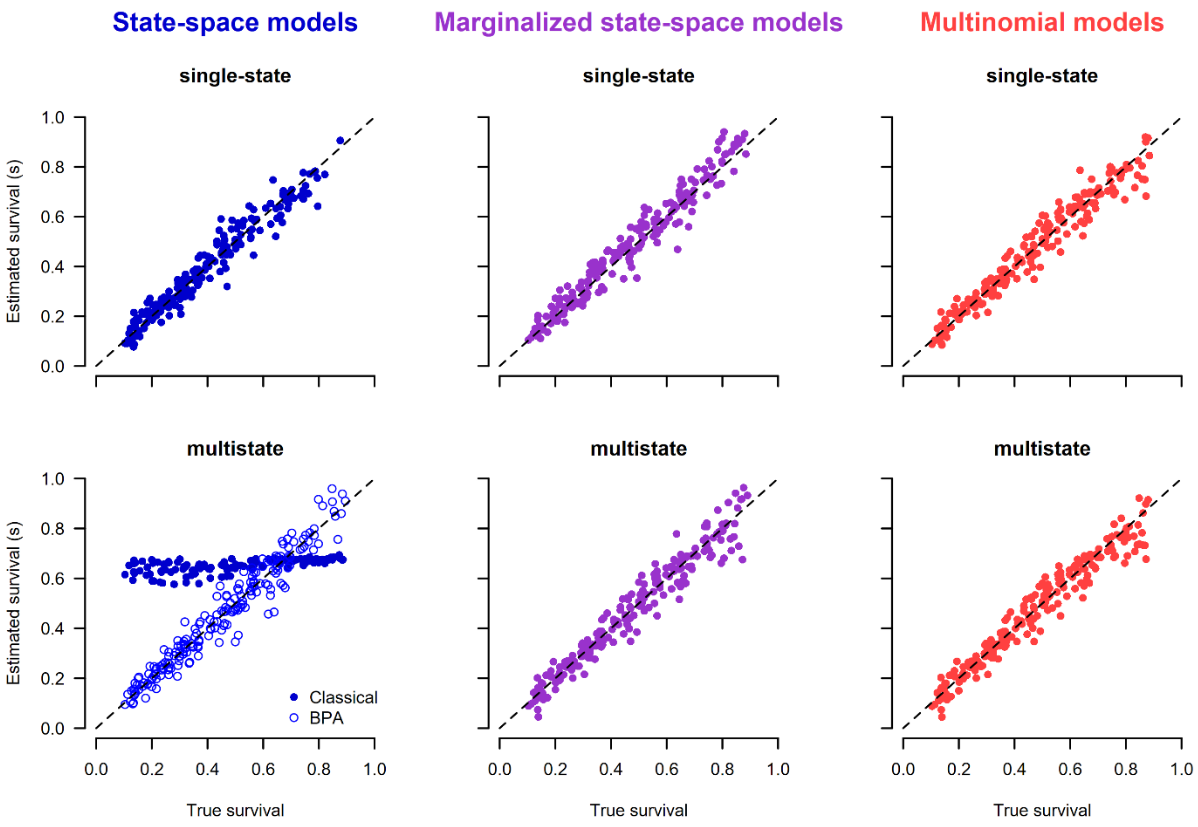

FIGURE S7. Scatterplots of posterior means versus the true values of survival obtained from different models analyzing dead-recovery data. The initial values for the latent state of the state-space models (left column) were generated assuming a random time of death. Each of the 200 converged simulation runs produced one point in the graphs.

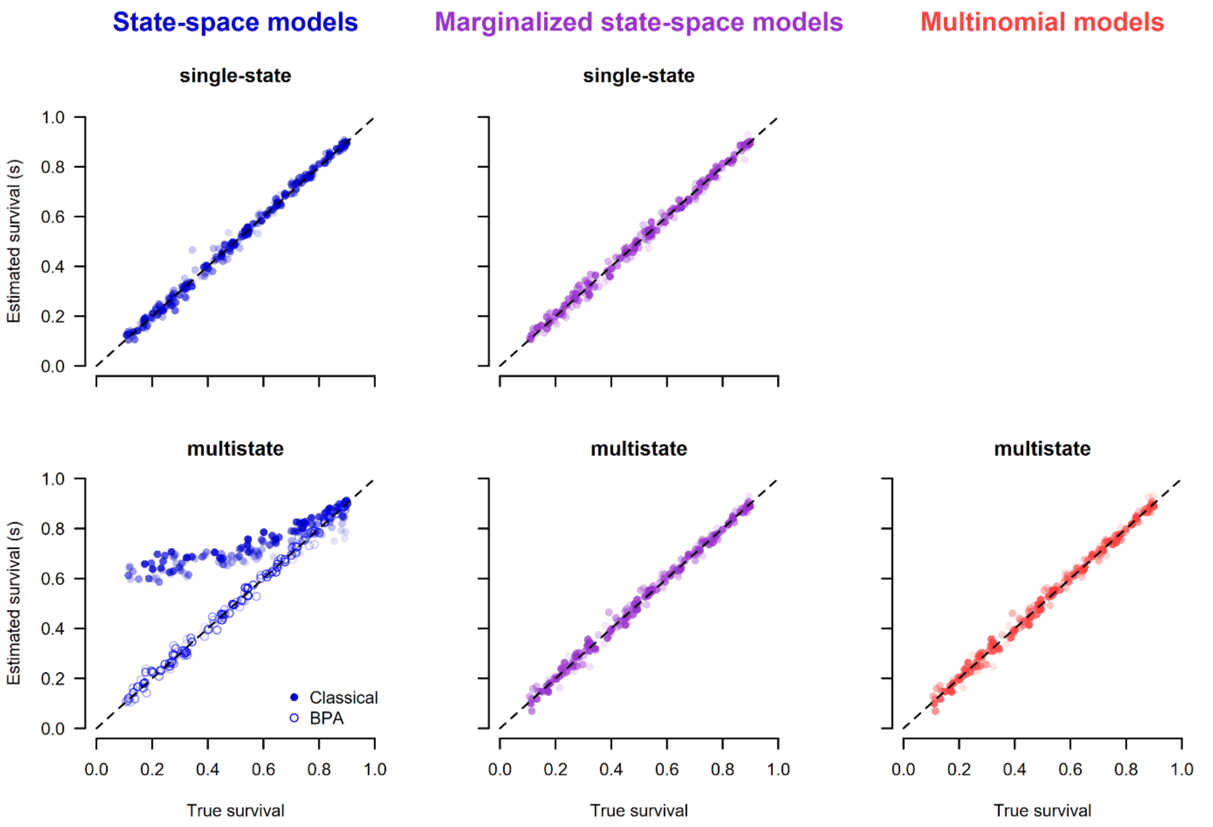

FIGURE S8. Scatterplots of posterior means versus the true values of survival obtained from different models jointly analyzing dead-recovery and live-encounter data. The initial values for the latent state of the state-space models (left column) were generated assuming a random time of death. The color gradient shows the values of the recapture probabilities used to simulate the data (the darker, the higher the probability of recapture). Each of the 200 converged simulation runs produced one point in the graphs.

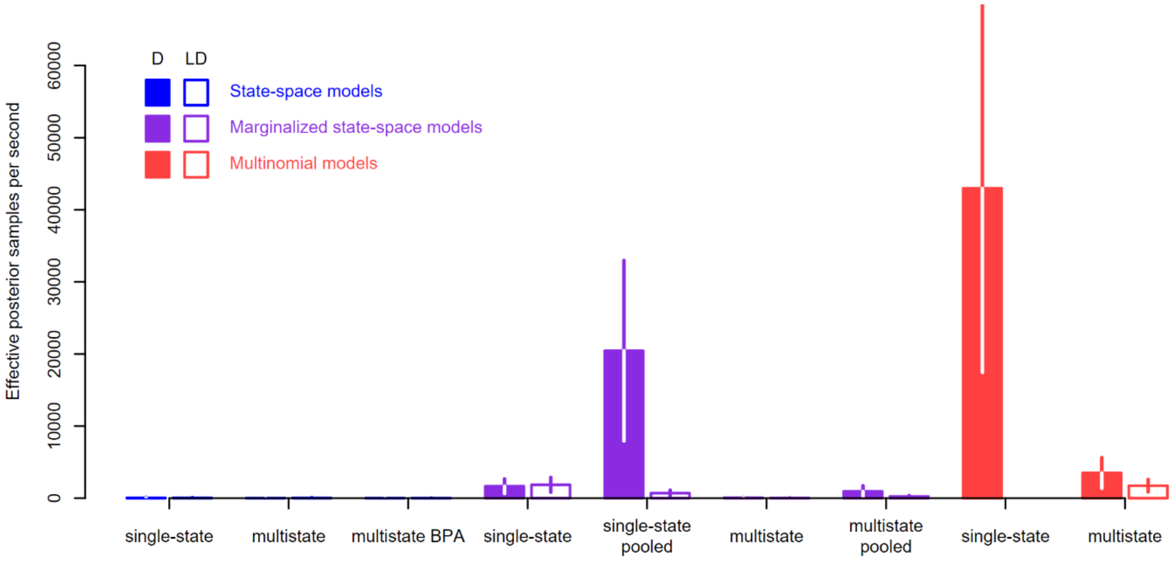

FIGURE S9. Computational efficiency of the different models measured as the number of independent effective posterior samples per second. The filled bars show the mean efficiency (across 10 data sets) of the dead-recovery models (D), the open bars show the mean efficiency of the models for the joint analysis of dead-recovery and live-encounter data (LD). The vertical lines show mean ± 1SD of efficiency. Note that the first three models on the left are state-space models (color hard to see).
